# Clinicopathologic analysis of microscopic tumor extension in glioma for external beam radiotherapy planning

**DOI:** 10.1186/s12916-021-02143-w

**Published:** 2021-11-17

**Authors:** Shulun Nie, Yufang Zhu, Jia Yang, Tao Xin, Song Xue, Jujie Sun, Dianbin Mu, Zhaoqiu Chen, Pengpeng Sun, Jinming Yu, Man Hu

**Affiliations:** 1 Shandong First Medical University and Shandong Academy of Medical Sciences, Qingdao Road 6699, Jinan, 250117 Shandong China; 2 Department of Radiation Oncology, Shandong Cancer Hospital and Institute, Shandong First Medical University and Shandong Academy of Medical Sciences, Jiyan Road 440, Jinan, 250117 Shandong China; 3 Department of Neurosurgery, Shandong Cancer Hospital and Institute, Shandong First Medical University and Shandong Academy of Medical Sciences, Jiyan Road 440, Jinan, 250117 Shandong China; 4 Department of Neurosurgery, The First Affiliated Hospital of Shandong First Medical University, Jingshi Road 16766, Jinan, 250013 Shandong China; 5 Department of Pathology, Shandong Cancer Hospital and Institute, Shandong First Medical University and Shandong Academy of Medical Sciences, Jiyan Road 440, Jinan, 250117 Shandong China; 6 Department of Radiology, Shandong Cancer Hospital and Institute, Shandong First Medical University and Shandong Academy of Medical Sciences, Jiyan Road 440, Jinan, 250117 Shandong China

**Keywords:** Glioma, Microscopic extension, Radiotherapy, Clinical target volume

## Abstract

**Background:**

There is no consensus regarding the clinical target volume (CTV) margins in radiotherapy for glioma. In this study, we aimed to perform a complete macropathologic analysis examining microscopic tumor extension (ME) to more accurately define the CTV in glioma.

**Methods:**

Thirty-eight supra-total resection specimens of glioma patients were examined on histologic sections. The ME distance, defined as the maximum linear distance from the tumor border to the invasive tumor cells, was measured at each section. We defined the CTV based on the relationships between ME distance and clinicopathologic features.

**Results:**

Between February 2016 and July 2020, a total of 814 slides were examined, corresponding to 162 slides for low-grade glioma (LGG) and 652 slides for high-grade glioma (HGG). The ME value was 0.69 ± 0.43 cm for LGG and 1.29 ± 0.54 cm for HGG (*P* < 0.001). After multivariate analysis, tumor grade, O^6^-methylguanine-DNA-methyltransferase promoter methylated status (MGMT_m_), isocitrate dehydrogenase wild-type status (IDH_wt_), and 1p/19q non-co-deleted status (non-codel) were positively correlated with ME distance (all *P* < 0.05). We defined the CTV of glioma based on tumor grade. To take into account approximately 95% of the ME, a margin of 1.00 cm, 1.50 cm, and 2.00 cm were chosen for grade II, grade III, and grade IV glioma, respectively. Paired analysis of molecularly defined patients confirmed that tumors that had all three molecular alterations (i.e., MGMT_m_/IDH_wt_/non-codel) were the most aggressive subgroups (all *P* < 0.05). For these patients, the margin could be up to 1.50 cm, 2.00 cm, and 2.50 cm for grade II, grade III, and grade IV glioma, respectively, to cover the subclinical lesions in 95% of cases.

**Conclusions:**

The ME was different between the grades of gliomas. It may be reasonable to recommend 1.00 cm, 1.50 cm, and 2.00 cm CTV margins for grade II, grade III, and grade IV glioma, respectively. Considering the highly aggressive nature of MGMT_m_/IDH_wt_/non-codel tumors, for these patients, the margin could be further expanded by 0.5 cm. These recommendations would encompass microscopic disease extension in 95% of cases.

**Trial registration:**

The trial was registered with Chinese Clinical Trial Registry (ChiCTR2100049376).

**Supplementary Information:**

The online version contains supplementary material available at 10.1186/s12916-021-02143-w.

## Background

Glioma, the most common malignant primary brain tumor in adults, is highly aggressive and associated with a poor prognosis [1]. Surgery is the first choice for the treatment of glioma. However, due to the diffusely infiltrating characteristics of glioma, the majority of patients do not benefit from surgery. In the past 20 years, technological developments in radiotherapy have made it a feasible option for maintaining good disease coverage and decreasing destruction to normal tissues. However, the optimal treatment volume for glioma remains a controversial issue. How far to extend the clinical target volume (CTV) beyond the gross tumor volume (GTV) is largely empiric and mainly left to the discretion of the physician [2]. The European Organisation for Research and Treatment of Cancer adopts a 2 cm volumetric expansion of the GTV to generate the CTV [3]. This is based on imaging data stating that more than 80% of recurrences occur within a 2 cm margin of the contrast-enhanced lesion on magnetic resonance (MR) imaging [4, 5]. The Radiation Therapy Oncology Group defines CTV as the residual tumor and resection cavity plus peritumoral brain edema (PTBE) enclosed by 2–2.5 cm margin [6], considering that findings have demonstrated high rates of glioma cells at PTBE areas [7, 8]. In the updated Version 1.2021 National Comprehensive Cancer Network (NCCN) guidelines, the CTV includes the visible lesion plus 1–2 cm margin [9]. Whether these arbitrary CTV margins are excessive or inadequate remains uncertain. The delineation of CTV in glioma must take into account the potential for microscopic extension (ME). Unfortunately, histopathology studies addressing the distribution of microscopic disease around glioma are sparse [7, 8, 10].

Therefore, the aim of this study was to quantitatively evaluate the ME of glioma from histologic investigations to define the appropriate CTV margins as precisely as possible. We also analyzed the association of ME with any particular clinicopathologic factors, including age, gender, tumor grade, tumor size, PTBE size, tumor location, O^6^-methylguanine-DNA-methyltransferase (MGMT) promoter methylation status, isocitrate dehydrogenase (IDH) mutation, and the co-deletion of chromosome arms 1p and 19q (1p/19q co-deletion), which would allow the CTV to be optimally adapted to individual situations.

## Methods

### Patients

In general, to meet the basic requirements of the linear regression model, the sample size (*n*) should be greater than 30, or *n* ≥ 3(*k* + 1), where *k* is the number of independent variables [11]. The preset number of independent variables in this study is 9 (*k* = 9), and the sample size *n* should be ≥ 30. Therefore, we sought an enrolled sample size of 30, and finally, 42 glioma patients treated at Shandong Cancer Hospital or the First Affiliated Hospital of Shandong First Medical University between February 2016 and July 2020 were recruited. The inclusion criteria were as follows: (a) patients aged 18 years or older, (b) preoperative Karnofsky Performance Status (KPS) ≥ 80, (c) tumor growth in non-functional areas, and (d) tumor location allowing supra-total resection and resection margins 2 cm and 3 cm from the tumor border (defined as the enhancing border of the tumor on T1-weighted MR imaging; for non-enhancing gliomas, tumor border was defined as the border of the hyperintensity signal seen on T2-weighted MR imaging). The exclusion criteria were as follows: (a) a history of a previous brain tumor, cranial surgery, radiotherapy, chemotherapy, or contraindication for MR imaging and (b) multifocal lesions. Intraoperative neuromonitoring and neuronavigation were performed to ensure safety and maximize the extent of intracranial tumor resection. The 2016 World Health Organization was used for postoperative grading [12]. This study was approved by two institutional review boards. Written informed consent was obtained from all participants.

### Preoperative MR image acquisition

Conventional gadolinium-enhanced T1-weighted (T1_ce_), T2-weighted (T2_w_), and T2-fluid attenuated inversion recovery (T2_FLAIR_) images were acquired in all patients before surgery. Acquisitions were performed using a 3T whole-body system (Philips Achieva 3.0T) with an 8-channel head coil. The MR scanning images were obtained parallel to the orbitomeatal line (OML). Imaging sequences included (a) T1_ce_ (repetition time/echo time [TR/TE] = 495/10 ms, slice thickness/gap = 3 mm/0 mm, number of signal averaged [NSA] = 1, field of view [FOV] = 260 mm × 260 mm, matrix = 256 × 256); (b) T2_w_ (TR/TE = 13312/110 ms, slice thickness/gap = 3 mm/0 mm, NSA = 1, FOV = 260 mm × 260 mm, matrix = 416 × 416); and (c) T2_FLAIR_ (TR/TE = 11000/120 ms, slice thickness/gap=3 mm/0 mm, NSA = 1, FOV = 260 mm × 260 mm, matrix = 320 × 320). MR imagings were analyzed and judged by two senior radiologists (ZQ.C. and PP.S.). In enhancing gliomas, the tumor-containing zone was defined as the area of increased signal intensity on T1_ce_ image. In non-enhancing gliomas, the tumor-containing zone was defined as the area of the FLAIR hyperintensity signal seen on the T2_w_ image. For all patients, the radiological tumor size was determined as the longest axis of the tumor in all three directions, measured by a radiologist (PP.S.) blinded with respect to the macroscopic tumor sizes.

### Sampling of tumor specimens

**Fig. 1:**
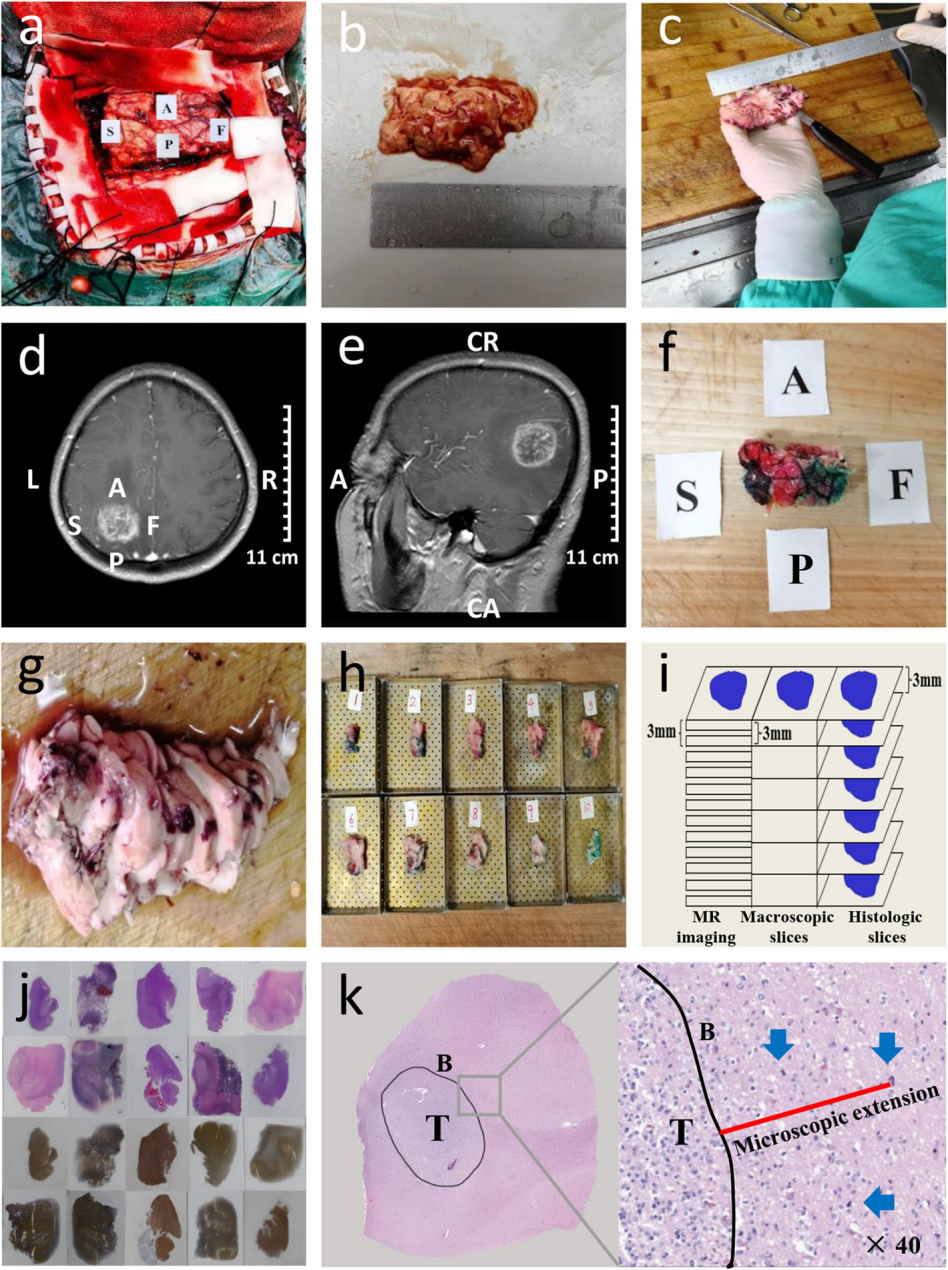
**a**–**j** Pathology procedure. First, the specimen was collected from surgery (**a**) and fixed in formalin. The dimensions of the specimen, both before and after fixation were recorded to determine the area retraction (**b**, **c**). Then, referring to the MR imaging (**d**, **e**), the edges of the specimen were inked with different colors to indicate the original orientation in the brain (**f**). Subsequently, the specimens were sectioned into 3 mm thick slices (**g**, **h**), which ensured that each specimen slice matched the MR slice (**i**). Finally, sections were embedded in paraffin and cut into 5 μm thick sections, which were stained with H&E and antibodies for immunohistochemistry (**j**). **k** Representative example of ME measurement. Left: Image of the slide without magnification. The tumor border was outlined in black. Right: Magnified H&E view (×40) shows invasive tumor cells (blue arrow) outside the primary tumor border. The red line represents the size of the microscopic tumor extension. A, anterior; P, posterior; S, skull; F, falx; L, left; R, right; CR, cranial; CA, caudal; T, tumor; B, tumor border

The process for large slice preparation was performed as described previously [13]. During surgery, the orientation of the excision specimen was marked with different alphabet tags (Fig. 1a). Meanwhile, the plane of the OML was marked on the specimen. Then, the specimen was fixed in 10% formalin for ≥ 24 h. The macroscopic tumor size, both before and after fixation, was recorded to determine the area retraction due to fixation (Fig. 1b, c and Additional file 1: Table S1). Next, referring to the preoperative MR imaging (Fig. 1d, e), the edges of the specimen were inked with different colors to indicate the original orientation of the specimen in the brain (Fig. 1f). We distinguished three directions: anterior-posterior, skull-falx, and cranio-caudal. Depending on the position of the tumor with respect to the brain, the skull-falx direction corresponds to the left-right direction in the patient. Subsequently, the plane of the OML was perpendicular to the table. The specimens were sectioned consecutively from the cranial side to the caudal side in 3 mm thick slices using a macrotome (Microm HM 450; GMI, Ramsey, Minnesota, USA) (Fig. 1g, h), which ensured that each specimen slice matched the MR imaging slice (Fig. 1i). Finally, sections were embedded in paraffin and cut into 5 μm thick sections, which were stained with hematoxylin and eosin (H&E) and subjected to immunohistochemistry to remark white matter (WM) fiber tracts (Fig. 1j). The following primary antibody was used: a mouse monoclonal antibody against S-100 (S1-61; Santa Cruz, California, USA; 1:100 dilution). An EnVision + System-HRP labeled polymer anti-mouse (Dako, California, USA) was used as the secondary antibody. Furthermore, molecular testing was performed in every patient. The details of the testing performed are included in the Additional file 2. Briefly, the methylation status of the MGMT promoter and the mutation of the IDH were identified using DNA pyro-sequencing. Deletion of chromosomal arms 1p and 19q was tested by use of fluorescence in- situ hybridization.

### Assessment of microscopic spread

**Fig. 2:**
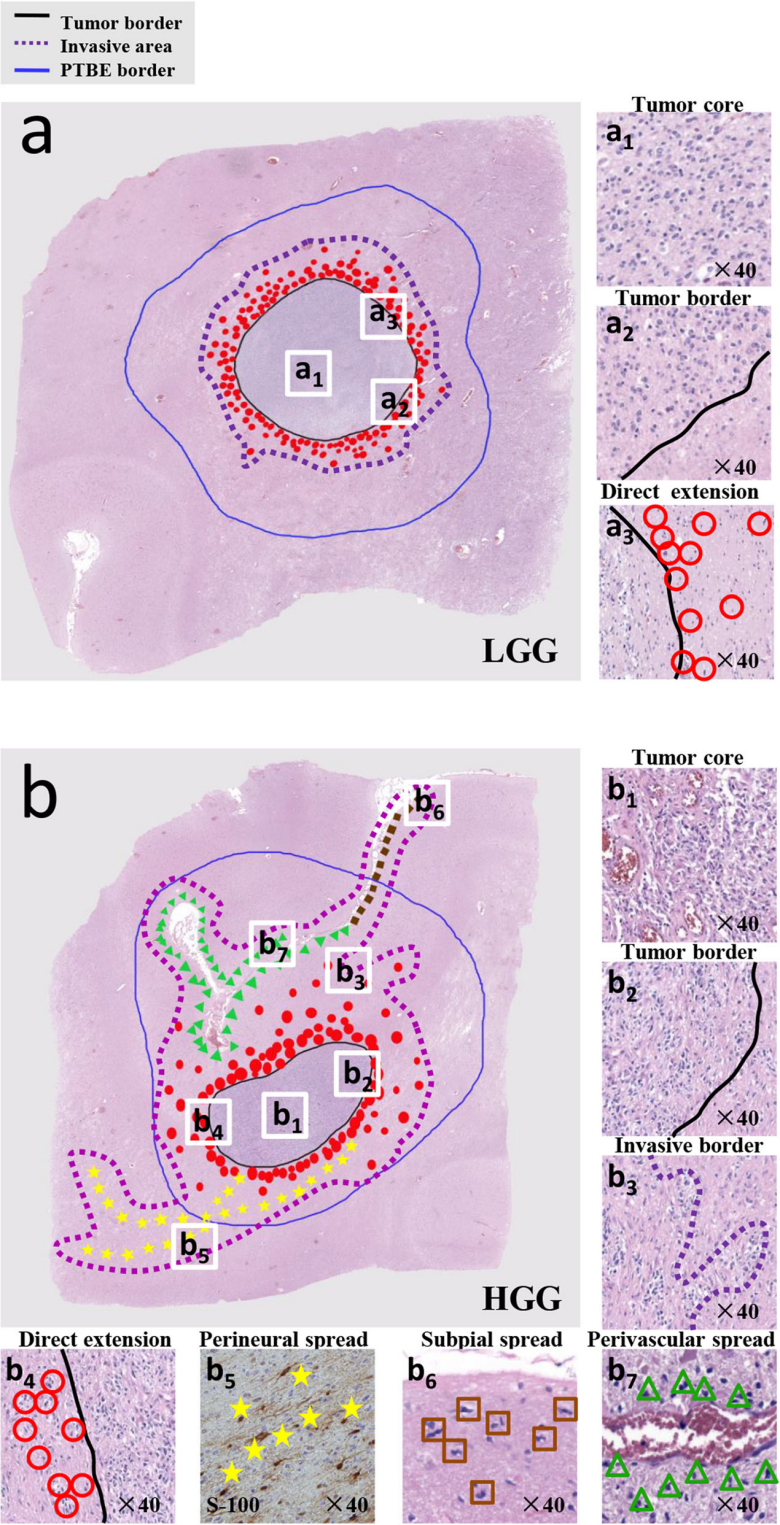
Pathology slices showing the invasive mode of gliomas of different grades. **a** LGG. **b** HGG. The red circles, yellow stars, brown squares, and green triangles represent direct extension, perineural spread, subpial spread, and perivascular spread, respectively. The black line delineates the tumor border, delimited by the naked eye, while the blue line delineates the PTBE border. The purple dotted line delineates the invasive area

To avoid interobserver variations, the same pathologist (JJ.S.) assessed the specimens and identified microscopic evidence of ME. First, the H&E sections were scanned by a confocal microscope (LSM800 with Airyscan 2; Carl Zeiss, Oberkochen, Germany). Considering the shrinkage of the surgical specimen, each scanning slide was correct through its corresponding scaling parameters (Additional file 1: Table S1). Then, on histologic slides, the tumor border and PTBE border were assessed with the naked eye (without magnification) and marked on its digitized version. The histologic tumor size and PTBE size, defined as the longest axis of the tumor and PTBE on slides, was also measured and recorded. Subsequently, we used a magnification of 10× to 40×, to search for glioma cells outside the tumor border. Invasive glioma cells were identified by means of their nuclear atypia, heteropyknotic staining, and elongated and hyperchromatic tumor nuclei [13]. Finally, we measured the ME distance relative to the edge of the invasive tumor bulk at each section. The ME was defined as the maximum linear distance from the tumor border to the farthest extent of the invasive tumor cells. An example of ME measured in a primary tumor is shown in Fig. 1k. A single investigator (DB.M.) performed all measurements. Furthermore, the mode of tumor extension was assessed according to the terms described by Louis et al. [14], classified according to four main types: (a) direct extension; (b) perineural spread, i.e., glioma cells grow along WM tracts; (c) subpial spread, i.e., tumor cells spread along pia mater; and (d) perivascular spread, defined by the presence of free tumor cells migrating along the basement membranes of blood vessels (Fig. 2).

### Statistical analysis

The difference between two groups was compared using the *Student’s t*-test, *Fisher's exact* test or *Chi-squared* test, as appropriate. Analysis of variance was used to investigate differences between groups numbering more than two. Post hoc analysis was used for pairwise comparisons. The relationship of radiologic size with macroscopic size and histology size was evaluated by *Spearman’s* rank correlation. Univariate and multivariate linear regression models were performed to determine independent factors associated with ME distance. To avoid multicollinearity, variance inflation factors for the variables were < 5. Statistical analyses were performed using SPSS Statistical software version 22.0 (IBM Armonk, New York, USA), and statistical significance was set at *P* < 0.05.

## Results

### Patient characteristics

Thirty-eight patients who met the eligibility criteria for this study were analyzed. The other four patients were eliminated either because the specimen was not well-fixed (*n* = 1) or not intact (*n* = 3). No patients experienced surgery-related complications. The clinical and pathological characteristics of the patients are displayed in Table 1. The patients included 21 men and 17 women, with a mean age of 48.5 years (range, 20–63 years). Imaging analysis showed that 8 patients had non-enhancing tumors (all had low-grade gliomas [LGG]), and 30 patients had enhancing tumors (all had high-grade gliomas [HGG]). HGG had a significantly larger PTBE size than LGG (mean ± standard deviation [SD], 2.22 ± 0.54 cm vs. 1.00 ± 0.45 cm, *P* < 0.001). In total, 162 slides of LGG derived from 8 grade II gliomas and 652 slides of HGG derived from 17 grade III gliomas and 13 grade IV gliomas were examined.

**Table 1:** Patient characteristics

Characteristic	LGG		HGG		Total		P**
	*n*	(%)	*n*	(%)	*n*	(%)	
Patients	8	(21)	30	(79)	38	(100)	
Slides*	162	(20)	652	(80)	814	(100)	
Age (years)							0.056
Mean (SD)	42	(7.52)	50.23	(11.07)	48.5	(10.87)	
Median (IQR)	41	(38–48.75)	54	(46.75–56.25)	50.5	(44.5–55)	
Gender							0.709
Male	5	(63)	16	(53)	21	(55)	
Female	3	(38)	14	(47)	17	(45)	
Tumor grade							< 0.001
Grade II	8	(100)	0	(0)	8	(21)	
Grade III	0	(0)	17	(57)	17	(45)	
Grade IV	0	(0)	13	(43)	13	(34)	
Histologic tumor size (cm)							0.126
Mean (SD)	2.94	(1.03)	3.72	(1.31)	3.56	(1.29)	
Median (IQR)	2.76	(2.27–3.65)	3.96	(2.69–4.72)	3.65	(2.43–4.67)	
Histologic PTBE size (cm)							< 0.001
Mean (SD)	1.00	(0.45)	2.22	(0.54)	1.96	(0.72)	
Median (IQR)	1.15	(0.48–1.35)	2.12	(1.84–2.86)	2.00	(1.45–2.43)	
Contrast enhancement							< 0.001
Yes	0	(0)	30	(100)	30	(79)	
No	8	(100)	0	(0)	8	(21)	
Lesion site							0.860
Frontal lobe	5	(63)	16	(53)	21	(55)	
Temporal lobe	2	(25)	11	(37)	13	(34)	
Occipital lobe	1	(13)	3	(10)	4	(11)	
MGMT methylation status							0.426
Unmethylated	5	(63)	12	(40)	17	(45)	
Methylated	3	(38)	18	(60)	21	(55)	
IDH mutation							0.698
Mutated	4	(50)	12	(40)	16	(42)	
Wild type	4	(50)	18	(60)	22	(58)	
1p/19q co-deletion							0.689
Co-deleted	2	(25)	11	(37)	13	(34)	
Non-co-deleted	6	(75)	19	(63)	25	(66)	

*Abbreviation: LGG* low-grade glioma; *HGG* high-grade glioma; *SD* standard deviation; *IQR* interquartile range; *PTBE* peritumoral brain edema; *MGMT* O^6^-methylguanine-DNA-methyltransferase; *IDH* isocitrate dehydrogenase; *1p/19q co-deletion*, the co-deletion of chromosome arms 1p and 19q

*Number of slides presenting this criterion

**P value according to the *Student’s* *t*-test or *Fisher's exact* test

### Radio-histologic correlations

**Fig. 3:**
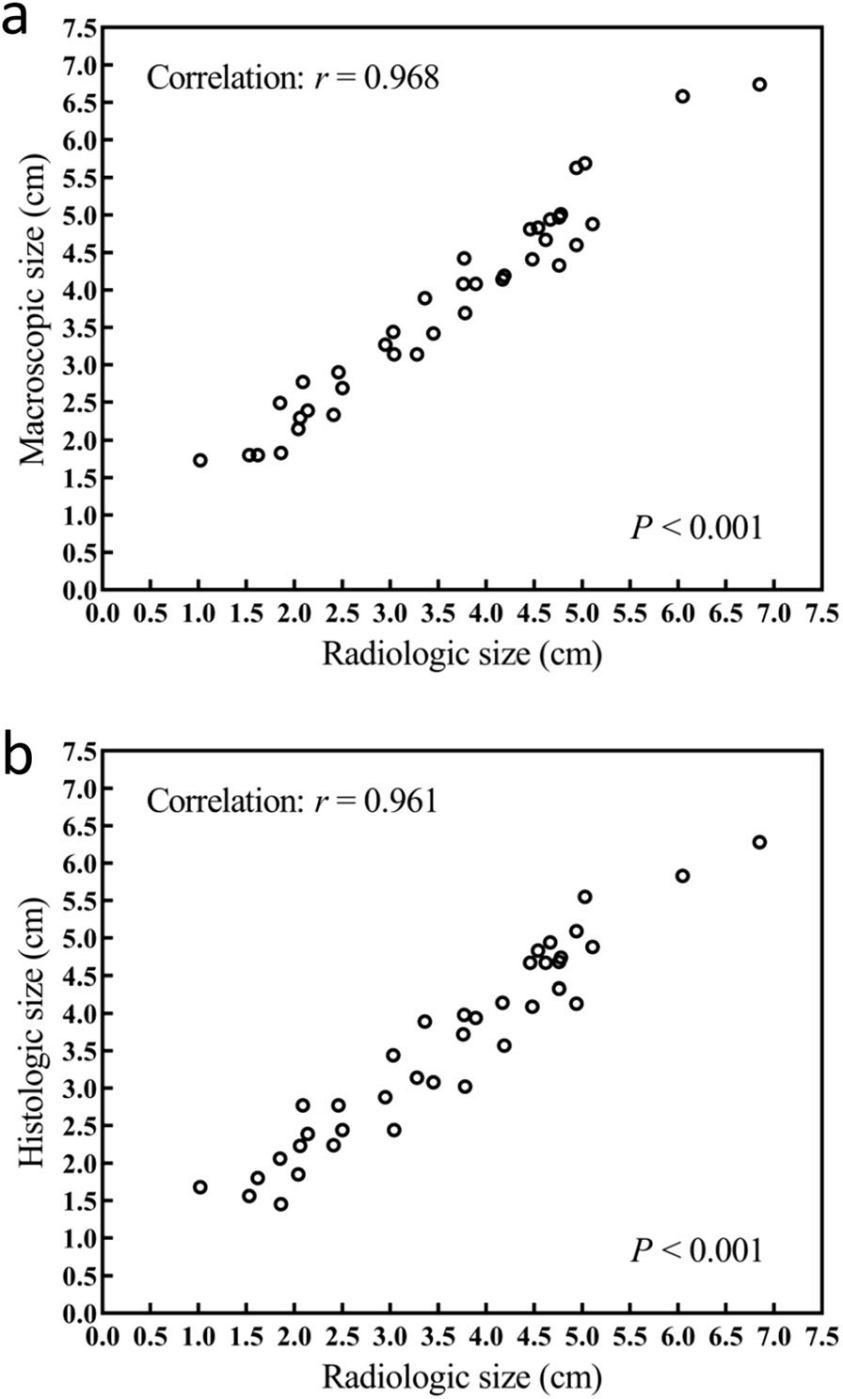
**a** Correlation between radiologic tumor size measured on MR imaging and macroscopic tumor size on all samples. **b** Correlation between radiologic tumor size measured on MR imaging and histologic tumor size on all samples

The histologic specimens of glioma were almost identical to their radiologic images in morphology. Further analysis revealed that the radiologic tumor size was smaller than its macroscopic size (mean ± SD, 3.59 ± 1.37 cm vs. 3.79 ± 1.33 cm, *P* < 0.001), but slightly larger than its histologic size (3.59 ± 1.37 cm vs. 3.56 ± 1.29 cm, *P* = 0.655). On MR imaging, the radiologic size of LGG was 2.95 ± 1.01 cm and of HGG was 3.76 ± 1.42 cm. HGG was larger than LGG, but the difference was not statistically significant (*P* = 0.140). This difference was also found on macroscopic and histologic measurements (Table 2). However, the most important finding was that comparative analysis of these three measurements revealed a significant correlation between radiologic tumor size and macroscopic tumor size (*r* = 0.968, *P* < 0.001) and histology tumor size (*r* = 0.961, *P* < 0.001) (Fig. 3). This was also found for enhancing and non-enhancing gliomas (all *P* < 0.001) (Additional file 3: Fig. S1). These findings demonstrated that it was reasonable to delineate the GTV of the glioma in the MR images.

**Table 2:** Measurement of radiologic, macroscopic, and histologic tumor dimensions for the same samples

	Mean (cm)	SD	Range (cm)	P*
Radiologic size				
LGG	2.95	1.01	1.86–4.78	0.140
HGG	3.76	1.42	1.02–6.85	
Macroscopic size				0.120
LGG	3.14	1.05	1.83–5.01	
HGG	3.97	1.35	1.73–6.74	
Histologic size				0.126
LGG	2.94	1.03	1.45–4.74	
HGG	3.72	1.31	1.56–6.28	

*Abbreviation: SD*, standard deviation; *LGG*, low-grade glioma; *HGG*, high-grade glioma

*P value according to the *Student’s* *t*-test

### Microscopic tumor extension

**Fig. 4:**
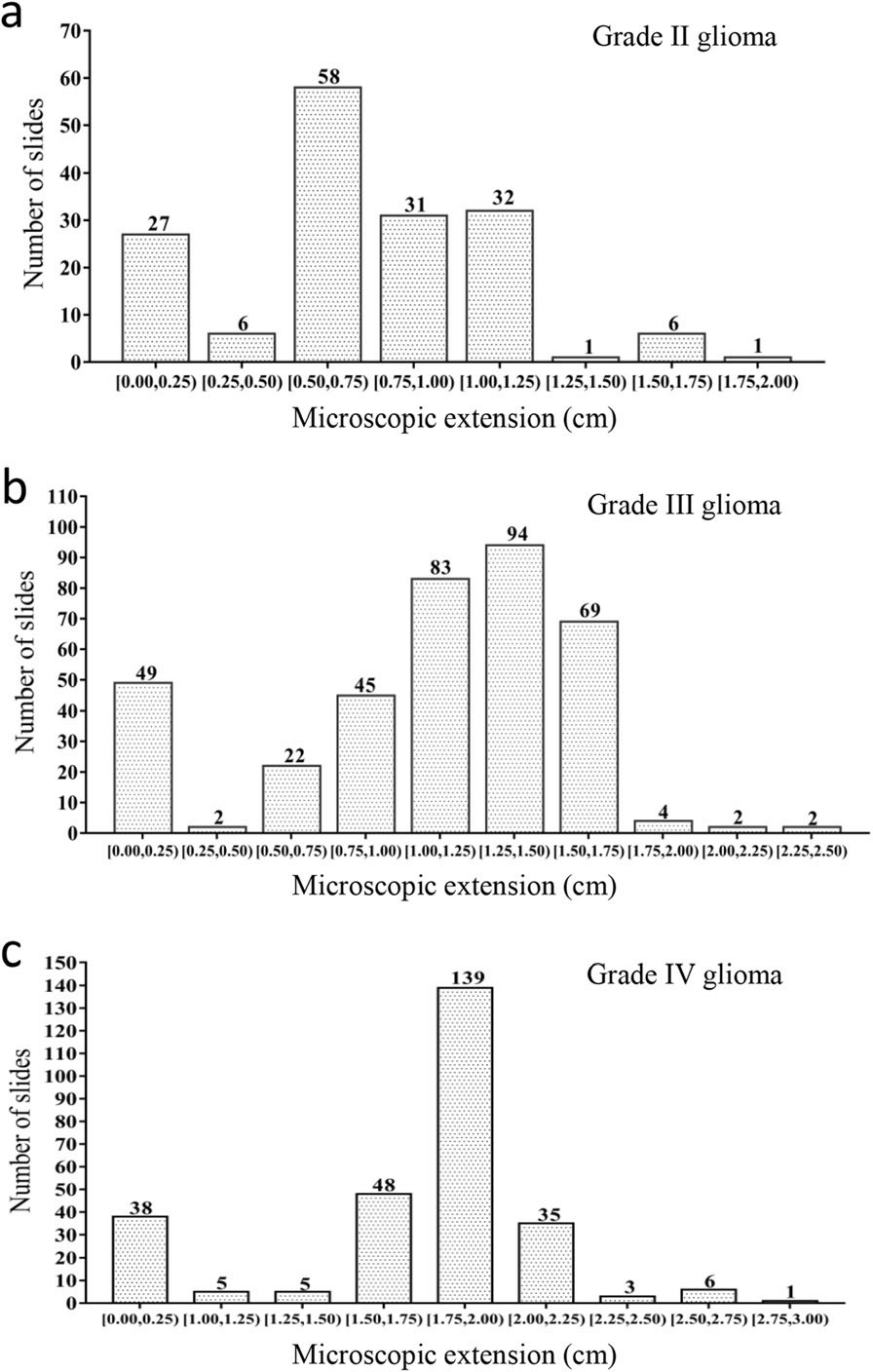
Microscopic extension distribution in different grades of gliomas. **a** Grade II gliomas; **b** Grade III gliomas; **c** Grade IV gliomas

The mean ME distance was 1.17 cm (range, 0 to 2.80 cm). We observed a significant difference in ME between LGG and HGG (*P* < 0.001), with a mean of 0.69 cm (range, 0 to 1.80 cm) for LGG, and 1.29 cm (range: 0 to 2.80 cm) for HGG. Further analysis showed that grade IV gliomas had a significantly higher ME than grade III and grade II tumors (mean ± SD, 1.59 ± 0.67 cm vs.1.06 ± 0.51 cm vs. 0.69 ± 0.37 cm, all *P* < 0.001). Analysis of the ME distribution confirmed the difference between the three histologic types (Tables 3, 4, and 5). Figure 4 presents ME frequency tables classified by increments of 0.25 cm together. Furthermore, we found that MGMT unmethylated tumors had a significantly lower ME than their methylated counterparts (1.07 ± 0.66 cm vs. 1.24 ± 0.61 cm, *P* < 0.001). In contrast, IDH wild-type tumors had a higher ME than IDH mutated tumors (1.32 ± 0.65 cm vs. 0.93 ± 0.55 cm, *P* < 0.001). Tumors with 1p/19q non-co-deletion had a higher ME than those with 1p/19q co-deletion (1.24 ± 0.68 cm vs. 1.02 ± 0.50 cm, *P* < 0.001) (Additional file 4: Fig. S2). In paired analysis, tumors that had all three alterations (i.e., MGMT methylated plus IDH wild-type plus 1p/19q non-co-deleted [MGMT_m_/IDH_wt_/non-codel]) showed a significantly higher ME than the other counterparts (all *P* < 0.05) (Additional file 5: Fig. S3).

**Table 3:** ME distribution for grade II glioma

ME (cm)	No.	Cumulative	No. %	Cumulative %
0.00	16	16	9.88	9.88
0.10	5	21	3.09	12.96
0.20	6	27	3.70	16.67
0.40	6	33	3.70	20.37
0.50	11	44	6.79	27.16
0.60	17	61	10.49	37.65
0.70	30	91	18.52	56.17
0.80	19	110	11.73	67.90
0.90	12	122	7.41	75.31
1.00	32	154	19.75	95.06
1.30	1	155	0.62	95.68
1.50	4	159	2.47	98.15
1.70	2	161	1.23	99.38
1.80	1	162	0.62	100

*Abbreviation: ME* microscopic extension; *No.* number of slides

**Table 4:** ME distribution for grade III glioma

ME (cm)	No.	Cumulative	No. %	Cumulative %
0.00	45	45	12.10	12.10
0.20	4	49	1.08	13.17
0.30	2	51	0.54	13.71
0.50	12	63	3.23	16.94
0.70	10	73	2.69	19.62
0.80	27	100	7.26	26.88
0.90	18	118	4.84	31.72
1.00	36	154	9.68	41.40
1.20	47	201	12.63	54.03
1.30	37	238	9.95	63.98
1.40	57	295	15.32	79.30
1.50	59	354	15.86	95.16
1.60	5	359	1.34	96.51
1.70	5	364	1.34	97.85
1.90	4	368	1.08	98.92
2.00	1	369	0.27	99.19
2.10	1	370	0.27	99.46
2.30	2	372	0.54	100

*Abbreviation: ME* microscopic extension; *No.* number of slides

**Table 5:** ME distribution for grade IV glioma

ME (cm)	No.	Cumulative	No. %	Cumulative %
0.00	38	38	13.57	13.57
1.00	2	40	0.71	14.29
1.20	3	43	1.07	15.36
1.30	1	44	0.36	15.71
1.40	4	48	1.43	17.14
1.50	14	62	5	22.14
1.60	13	75	4.64	26.79
1.70	21	96	7.5	34.29
1.80	62	158	22.14	56.43
1.90	77	235	27.5	83.93
2.00	31	266	11.07	95
2.20	4	270	1.43	96.43
2.30	3	273	1.07	97.5
2.50	1	274	0.36	97.86
2.60	4	278	1.43	99.29
2.70	1	279	0.36	99.64
2.80	1	280	0.36	100

*Abbreviation: ME* microscopic extension; *No.* number of slides

Figure 2 and Additional file 6: Table S2 show the invasion mode between different grades of gliomas. Direct extension (68%) was the most frequent pattern observed for all groups, followed by perineural spread (12%) and subpial growth (11%). Furthermore, compared with LGG, more HGG tumor cells infiltrated through perineural dissemination (13% vs. 4%, *P* = 0.001) and subpial growth (13% vs. 5%, *P* = 0.006).

#### PTBE

PTBE infiltration was found in 76% (615/814) of slides, corresponding to 106 slides for LGG, and 509 slides for HGG. Compared with LGG, PTBE infiltration was associated with more HGG (78% vs. 65%, *P* = 0.001). However, further analysis showed that the majority of PTBE infiltration occurred only at the margin of the primary tumor (Fig. 2a), and the extent of ME was much smaller than the PTBE size (mean ± SD, 1.52 ± 0.34 cm vs. 2.41 ± 0.52 cm, *P* < 0.001). In contrast, tumor cell infiltration extended beyond the PTBE area in 34% (277/814) of slides, including 73 slides for LGG, and 204 slides for HGG. In the brain parenchyma, outside PTBE infiltration occurred when tumor cells migrated along WM tracts and pia mater (Fig. 2b). Linear regression analysis revealed no significant relationship between ME distance and PTBE size (*P* = 0.122) (Table 6).

**Table 6:** Variables associated with ME distance in univariate and multivariate linear regression model

Variables	Univariate analysis		Multivariate analysis		
	*β* (95% CI)	*P* value	*β* (95% CI)	*P* value	VIF
Age (years)	0.004 (−0.014 to 0.021)	0.652			
Gender					
Male/female	0.150 (−0.225 to 0.526)	0.421			
Tumor grade					
LGG/HGG	0.884 (0.532–1.236)	< 0.001	0.822 (0.584–1.060)	< 0.001	1.078
Histologic tumor size (cm)	0.092 (−0.053 to 0.237)	0.208			
Histologic PTBE size (cm)	0.199 (−0.056 to 0.454)	0.122			
Tumor location					
Frontal lobe	Ref.	Ref.			
Temporal lobe	−0.133 (−0.528 to 0.261)	0.497			
Occipital lobe	−0.388 (−0.987 to 0.212)	0.198			
Molecular markers					
MGMT methylation status					
Unmethylated/Methylated	0.456 (0.110–0.802)	0.011	0.259 (0.066–0.453)	0.010	1.060
IDH mutation					
Mutated/wild type	0.617 (0.298–0.936)	< 0.001	0.391 (0.168–0.613)	0.001	1.382
1p/19q co-deletion					
Co-deleted/non-co-deleted	0.472 (0.109–0.836)	0.012	0.300 (0.066–0.534)	0.013	1.412

*Abbreviation: ME* microscopic extension; *β* regression coefficient; *CI* confidence interval; *VIF* variance inflation factor; *LGG* low-grade glioma; *HGG* high-grade glioma; *PTBE* peritumoral brain edema; *MGMT* O^6^-methylguanine-DNA-methyltransferase; *IDH* isocitrate dehydrogenase; *1p/19q co-deletion* the co-deletion of chromosome arms 1p and 19q

#### Definition of optimal CTV margins in glioma

**Fig. 5:**
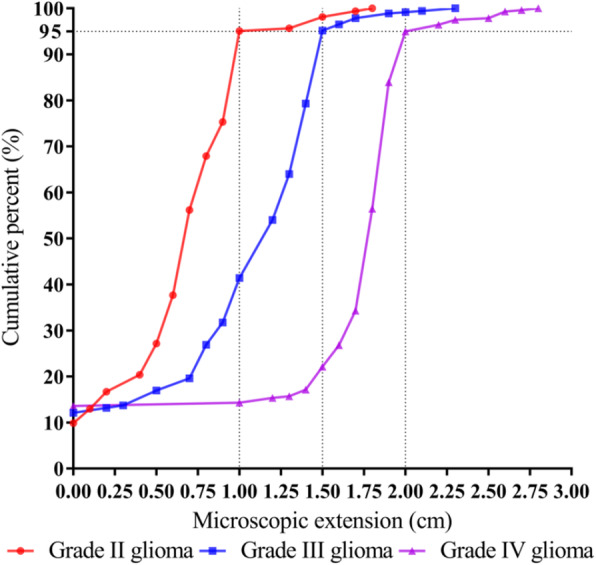
Cumulative percentage of the sample as a function of the extent of microscopic extension by tumor grade

The detailed results of univariate and multivariate analyses are displayed in Table 6. Clinical factors associated with the ME distance included tumor grade, MGMT methylation status, IDH mutation, and 1p/19q co-deletion (all *P* < 0.05). In multivariate analysis, each factor remained significantly and independently associated with ME distance, with tumor grade having the largest β-coefficient (0.822). Therefore, we defined the CTV of glioma based on grade. Using an approximately 95% probability to cover the ME, a margin for grade II gliomas corresponded to 1.00 cm, whereas for grade III and IV gliomas, this margin was 1.50 cm and 2.00 cm, respectively (Tables 3, 4, and 5 and Fig. 5). Considering the highly aggressive nature of MGMT_m_/IDH_wt_/non-codel tumors, we performed a subgroup analysis of this subtype of tumor. The analysis results are shown in Additional file 7: Table S3 and Fig. S4. For these types of tumors, we suggested that the margin could be up to 1.50 cm, 2.00 cm, and 2.50 cm for grade II, grade III, and grade IV glioma, respectively, to fully cover the subclinical lesions in 95% of cases.

## Discussion

Microscopic tumor extension is regarded as the “gold standard” to accurately define the CTV for radiotherapy. A quantitative pathologic evaluation of subclinical invasion from the primary lesion into surrounding tissues for planning external beam radiotherapy has been performed in some tumor sites, including cervix carcinoma, breast cancer, hepatocellular carcinoma, thyroid cancer, and lung cancer [15–19]. However, to date, there are minimal data available for glioma [7, 10], which has resulted in the lack of uniform guidelines to guide CTV delineation in glioma, and each radiotherapy center varies greatly [2, 3, 6, 9].

To our knowledge, this is the first prospective study to analyze the macropathology of glioma and to quantify ME beyond identifiable GTV margins. To validate our methodology, we first examined the relationship between pathologic findings and imaging findings. We demonstrated that the radiologic tumor size measured on preoperative MR imaging was closely related to its macroscopic size and histologic size, delimited by the naked eye. This was also found for both enhancing and non-enhancing gliomas. This suggests that the tumor border defined on MR imaging is only the gross tumor boundary, which does not represent the true boundary of tumor extension. This is consistent with the findings of previous studies [20, 21]. This result provides useful evidence for the delineation of the GTV of glioma based on MR imaging. In terms of ME of tumors, the present study determined that ME was heterogeneously distributed in different glioma individuals, which is related to tumor grade and MGMT methylation, IDH mutation, and 1p/19q co-deletion status. It is noteworthy that tumor grade was the most important factor that had a significant impact on ME distance. In our study, HGG showed more extensive infiltration of the normal brain than LGG, which may be related to the stronger proliferation activity and greater invasiveness of HGG tumor cells [22, 23]. We defined the CTV of glioma based on tumor grade. It may be reasonable to recommend 1.00 cm, 1.50 cm, and 2.00 cm CTV margins for grade II, grade III, and grade IV glioma, respectively, to cover the subclinical lesions in 95% of cases.

MGMT promoter methylation status is associated with the recurrence pattern of glioma. Two previous studies [24, 25] reported that patients with MGMT methylated glioblastomas have a higher tendency to develop distant recurrence than those without methylation. These findings were supported by the results of our study, in which MGMT methylation showed increased tumor migration and invasion than unmethylated tumors. These data may explain why distant failure was more frequent in methylated tumors than in unmethylated tumors. Moreover, we observed that IDH wild-type tumors contribute to more aggressive infiltration than their mutated counterparts, leading to extensive ME. The NCCN guidelines recommend radiotherapy dose escalation for IDH wild-type gliomas, as these patients have a more aggressive course of disease [9]. Our results support the evidence of the guidelines from a histopathological perspective. Interestingly, the present study also showed that 1p/19q non-co-deleted tumors had a higher ME extension than those with co-deleted, which has not been described before. Future studies could use bioassays to assess invasive propensity to explain these results. In order to find the most aggressive molecular combination, we defined eight groups with the use of the above molecular markers (Additional file 5: Fig. S3). Finally, tumors that had all three molecular alterations (i.e., MGMT_m_/IDH_wt_/non-codel) were proved to be the most aggressive subtype. For these types of tumors, we suggested that the margin reach 1.50 cm, 2.00 cm, and 2.50 cm for grade II, grade III, and grade IV glioma, respectively, to cover 95% of the ME.

In this series, no correlation was found between ME and tumor size in glioma, which is consistent with the study focusing on the extent of ME in hepatocellular carcinoma [17]. These correlations of ME distance with the histopathological features of the tumor and not the tumor size imply that ME may be related to the biological characteristics and aggressiveness of the primary tumor [17].

PTBE is a frequently encountered phenomenon in glioma and is considered significantly associated with the clinical outcome of patients [26, 27]. However, due to the unknown relationship of the invasive extent of tumor cells with PTBE, the necessity of irradiating PTBE in radiotherapy remains controversial. In our study, the ME of glioma and PTBE did not show a correlation. We found a large number of aggressive tumor cells in the PTBE area, which is consistent with the results from previous histological studies [7, 8]. However, the majority of PTBE infiltration occurred only at the margin of the primary tumor. We did not find that the wider the edema area was, the wider the range of glioma cell infiltration. In contrast, the extent of ME was much smaller than that of PTBE zone. Furthermore, in 34% of slices, outside PTBE infiltration occurred when tumor cells migrated along WM fiber tracts and pia mater. Therefore, we think that radiotherapy including PTBE was unreasonable.

Tumor invasion is a complex process that depends on the interactions between tumor cells and peripheral normal tissue [28]. In our study, glioma cells followed two main pathways to invade adjacent tissue. Direct extension is the main pathway of extension of tumor cells to adjacent sites. This infiltration mode accounted for a large proportion of both LGG and HGG. The second pathway is local metastases. In the brain parenchyma, this extension occurs along WM tracts, pia mater, and basement membranes of blood vessels. Although this pathway accounts for a small percentage, it is noteworthy that perineural spread was found to be effectively associated with a greater value of ME in previous study [28]. The existence of WM tracts allows rapid migration of tumor cells well beyond the peripheral region of the primary tumor [28]. Detailed pathologic studies have shown selective infiltration by tumor cells along the neural tracts rather than isotropic infiltration (Fig. 2b). ME is not uniform in all dimensions, which results in anisotropic changes at the edge of the CTV. Diffusion tensor imaging (DTI) is widely used to visualize WM tracts and can be used as a noninvasive imaging method to assess peritumoral involvement in WM tracts [29–31]. Future studies can combine DTI with macropathology to perform individualized target volume delineation.

However, a few limitations in our study should be mentioned. First, the presented strategies to define the CTV margins are based on an analysis of 38 patients, which was a relatively small population. In order to validate the efficacy and safety of the CTV margin determined based on ME (CTV_ME_), we performed a retrospectively study (Additional file 8). Although the outcome of patients is improved with the use of CTV_ME_ radiotherapy compared to non-CTV_ME_ radiotherapy, the difference was not statistically significant. But the results should be interpreted with care. Retrospective study might have been influenced by unrecognized biases. Our analyses were not controlled for confounding factors. Some factors, such as the molecular status, preoperative KPS, treatment regimens, extent of resection, etc., may exist and require further analysis. Therefore, future prospective studies are necessary to evaluate the CTV_ME_ performance for radiotherapy. Second, the slides of specimens were only representative of two dimensional sections of the resected tumor, which may not truly illustrate the ME in three dimensions. Some ME measurements may have been slightly underestimated. Third, the CTV defined in the present study is only the isotropic margin. As the present study and Unkelbach et al. [32] found, in some patients, the anisotropy of glioma extent is not determined by a single margin value. Future studies should combine DTI with macropathology to help with the design of individualize margins.

## Conclusions

It was reasonable to delineate GTV based on MR imaging. The GTV of glioma should include the surgical cavity (if present) plus any residual enhancing tumor on T1_ce_ MR imaging (in non-enhancing gliomas, consideration may be given to include T2_FLAIR_ abnormality in the GTV), without inclusion of PTBE. In terms of CTV, the ME of glioma is related to tumor grade and MGMT methylation, IDH mutation and 1p/19q co-deletion status. According to the histopathologic results of our ME investigation, it is reasonable to recommend 1.00 cm, 1.50 cm, and 2.00 cm CTV margins for grade II, grade III, and grade IV glioma, respectively. Considering the highly aggressive MGMT_m_/IDH_wt_/non-codel tumors, for these patients, the margin could be further expanded by 0.5 cm. These recommendations would adequately cover microscopic disease extension in 95% of cases. Future work will focus on combined imaging and pathology to help with the design of individualized target volume margins.

## Supplementary Information

**Additional file 1: Table S1.** Macroscopic tumor size before and after fixation, corresponding area retraction ratio for each case.

**Additional file 2:.** Molecular testing methods. (a) MGMT promoter methylation. (b) IDH1 mutations. (c) Detection of 1p/19q co-deletion.

**Additional file 3: Fig. S1.** (a) Correlation between radiologic tumor size measured on MR imaging and macroscopic tumor size on enhancing gliomas. (b) Correlation between radiologic tumor size measured on MR imaging and histologic tumor size on enhancing gliomas. (c) Correlation between radiologic tumor size measured on MR imaging and macroscopic tumor size on non-enhancing gliomas. (d) Correlation between radiologic tumor size measured on MR imaging and histologic tumor size on non-enhancing gliomas.

**Additional file 4: Fig. S2.** Histogram analysis showing the microscopic extension in different subgroups. (a) Tumor grade. (b) MGMT promoter methylation status. (c) IDH mutation status. (d) 1p/19q co-deletion status.

**Additional file 5: Fig. S3.** Histogram analysis showing the microscopic extension in different molecular groups.

**Additional file 6: Table S2.** The invasion mode between different grades of gliomas**.**

**Additional file 7: Table S3.** (a) ME distribution for grade II glioma in triple-positive molecular group. (b) ME distribution for grade III glioma in triple-positive molecular group. (c) ME distribution for grade IV glioma in triple-positive molecular group. **Fig. S4.** Cumulative distribution of ME for different grades gliomas in triple-positive molecular group**.**

**Additional file 8: **CTV performance validation. **Table S4.** Baseline characteristics of validation cohort. **Fig. S5.** Comparison of overall survival and progression-free survival in all patients and different grades of gliomas.

## Data Availability

The datasets used and analyzed during the current study are available from the corresponding author on reasonable request.

## Abbreviations

1p/19q co-deletion: The co-deletion of chromosome arms 1p and 19q
CTV: Clinical target volume
DTI: Diffusion tensor imaging
FLAIR: Fluid attenuated inversion recovery
FOV: Field of view
GTV: Gross tumor volume
H&E: Hematoxylin and eosin
HGG: High-grade glioma
IDH: Isocitrate dehydrogenase
KPS: Karnofsky performance status
LGG: Low-grade glioma
ME: Microscopic extension
MGMT: O^6^-Methylguanine-DNA-methyltransferase
MR: Magnetic resonance
NCCN: National comprehensive cancer network
NSA: Number of signal averaged
OML: Orbitomeatal line
PTBE: Peritumoral brain edema
SD: Standard deviation
TE: Echo time
TR: Repetition time
WM: White matter

## References

[1] Kruchko C, Ostrom QT, Gittleman H, Barnholtz-Sloan JS. The CBTRUS story: providing accurate population-based statistics on brain and other central nervous system tumors for everyone. Neuro Oncol. 2018; 20(3):295-298. 10.1093/neuonc/noy006. 10.1093/neuonc/noy006 PMC5817957 29471448

[2] Niyazi M, Brada M, Chalmers AJ, Combs SE, Erridge SC, Fiorentino A, Grosu AL, Lagerwaard FJ, Minniti G, Mirimanoff RO, Ricardi U, Short SC, Weber DC, Belka C ESTRO-ACROP guideline “target delineation of glioblastomas”. Radiother Oncol. 2016; 118(1):35-42. 10.1016/j.radonc.2015.12.003. 10.1016/j.radonc.2015.12.003 26777122

[3] Stupp R, Hegi ME, Gorlia T, Erridge SC, Perry J, Hong YK, Aldape KD, Lhermitte B, Pietsch T, Grujicic D, Steinbach JP, Wick W, Tarnawski R, Nam DH, Hau P, Weyerbrock A, Taphoorn MJ, Shen CC, Rao N, Thurzo L, Herrlinger U, Gupta T, Kortmann RD, Adamska K, McBain C, Brandes AA, Tonn JC, Schnell O, Wiegel T, Kim CY, Nabors LB, Reardon DA, van den Bent M, Hicking C, Markivskyy A, Picard M, Weller M, European Organisation for Research and Treatment of Cancer (EORTC)., Canadian Brain Tumor Consortium., CENTRIC study team. Cilengitide combined with standard treatment for patients with newly diagnosed glioblastoma with methylated MGMT promoter (CENTRIC EORTC 26071-22072 study): a multicentre, randomised, open-label, phase 3 trial. Lancet Oncol. 2014; 15(10):1100-1108. 10.1016/s1470-2045(14)70379-1. 10.1016/S1470-2045(14)70379-1 25163906

[4] Chang EL, Akyurek S, Avalos T, Rebueno N, Spicer C, Garcia J, Famiglietti R, Allen PK, Chao KSC, Mahajan A, Woo SY, Maor MH Evaluation of peritumoral edema in the delineation of radiotherapy clinical target volumes for glioblastoma. Int J Radiat Oncol Biol Phys. 2007; 68(1):144-150. 10.1016/j.ijrobp.2006.12.009. 10.1016/j.ijrobp.2006.12.009 17306935

[5] Aydin H, Sillenberg I, von Lieven H. Patterns of failure following CT-based 3-D irradiation for malignant glioma. Strahlenther Onkol. 2001; 177(8):424-431. 10.1007/pl00002424. 10.1007/pl00002424 11544905

[6] Chinot OL, Wick W, Mason W, Henriksson R, Saran F, Nishikawa R, Carpentier AF, Hoang-Xuan K, Kavan P, Cernea D, Brandes AA, Hilton M, Abrey L, Cloughesy T Bevacizumab plus radiotherapy-temozolomide for newly diagnosed glioblastoma. N Engl J Med. 2014; 370(8):709-722. 10.1056/NEJMoa1308345. 10.1056/NEJMoa1308345 24552318

[7] Yamahara T, Numa Y, Oishi T, Kawaguchi T, Seno T, Asai A, Kawamoto K. Morphological and flow cytometric analysis of cell infiltration in glioblastoma: a comparison of autopsy brain and neuroimaging. Brain Tumor Pathol. 2010; 27(2):81-87. 10.1007/s10014-010-0275-7. 10.1007/s10014-010-0275-7 21046309

[8] Eidel O, Burth S, Neumann JO, Kieslich PJ, Sahm F, Jungk C, Kickingereder P, Bickelhaupt S, Mundiyanapurath S, Bäumer P, Wick W, Schlemmer HP, Kiening K, Unterberg A, Bendszus M, Radbruch A Tumor infiltration in enhancing and non-enhancing parts of glioblastoma: a correlation with histopathology. PLoS One. 2017; 12(1):e0169292. 10.1371/journal.pone.0169292. 10.1371/journal.pone.0169292 PMC5245878 28103256

[9] National Comprehensive Cancer Network. (NCCN) Clinical Practice Guidelines in Oncology. Central Nervous System Cancers, Version 1. 2021. https://www.nccn.org/professionals/physician_gls/f_guidelines.asp. Accessed 4 Jun 2021. In.

[10] Mangiola A, de Bonis P, Maira G, Balducci M, Sica G, Lama G, Lauriola L, Anile C Invasive tumor cells and prognosis in a selected population of patients with glioblastoma multiforme. Cancer. 2008; 113(4):841-846. 10.1002/cncr.23624. 10.1002/cncr.23624 18618580

[11] Field A (2013). Everything you never wanted to know about statistics. Discovering Statistics Using IBM SPSS Statistics: And Sex and Drugs and Rock ‘n’ Roll.

[12] Louis DN, Perry A, Reifenberger G, von Deimling A, Figarella-Branger D, Cavenee WK, Ohgaki H, Wiestler OD, Kleihues P, Ellison DW The 2016 World Health Organization Classification of Tumors of the Central Nervous System: a summary. Acta Neuropathol. 2016; 131(6):803-820. 10.1007/s00401-016-1545-1. 10.1007/s00401-016-1545-1 27157931

[13] Nie S, Zhu Y, Yang J, Xin T, Xue S, Zhang X, Sun J, Mu D, Gao Y, Chen Z, Ding X, Yu J, Hu M Determining optimal clinical target volume margins in high-grade glioma based on microscopic tumor extension and magnetic resonance imaging. Radiat Oncol. 2021; 16(1):97. 10.1186/s13014-021-01819-0. 10.1186/s13014-021-01819-0 PMC8186169 34098965

[14] Louis DN. Molecular pathology of malignant gliomas. Annu Rev Pathol. 2006; 1:97-117. 10.1146/annurev.pathol.1.110304.100043, 1. 10.1146/annurev.pathol.1.110304.100043 18039109

[15] Xie WJ, Wu X, Xue RL, Lin XY, Kidd EA, Yan SM, Zhang YH, Zhai TT, Lu JY, Wu LL, Zhang H, Huang HH, Chen ZJ, Li DR, Xie LX More accurate definition of clinical target volume based on the measurement of microscopic extensions of the primary tumor toward the uterus body in international federation of gynecology and obstetrics Ib-IIa squamous cell carcinoma of the cervix. Int J Radiat Oncol Biol Phys. 2015; 91(1):206-212. 10.1016/j.ijrobp.2014.09.009. 10.1016/j.ijrobp.2014.09.009 25442332

[16] Nguyen BT, Deb S, Fox S, Hill P, Collins M, Chua BH. A prospective pathologic study to define the clinical target volume for partial breast radiation therapy in women with early breast cancer. Int J Radiat Oncol Biol Phys. 2012; 84(5):1116-1122. 10.1016/j.ijrobp.2012.02.038. 10.1016/j.ijrobp.2012.02.038 22543203

[17] Wang W, Feng X, Zhang T, Jin J, Wang S, Liu Y, Song Y, Liu X, Yu Z, LI Y. Prospective evaluation of microscopic extension using whole-mount preparation in patients with hepatocellular carcinoma: Definition of clinical target volume for radiotherapy. Radiat Oncol. 2010; 5:73. 10.1186/1748-717x-5-73, 1. 10.1186/1748-717X-5-73 PMC2936917 20731853

[18] Nixon IJ, Ganly I, Patel S, Palmer FL, Whitcher MM, Tuttle RM, Shaha AR, Shah JP The impact of microscopic extrathyroid extension on outcome in patients with clinical T1 and T2 well-differentiated thyroid cancer. Surgery. 2011; 150(6):1242-1249. 10.1016/j.surg.2011.09.007. 10.1016/j.surg.2011.09.007 PMC4151609 22136847

[19] Meynard C, Mansuet-Lupo A, Giraud N, Boulle G, Imbault P, Guénégou-Arnoux A, Bobbio A, Durdux C, Damotte D, Giraud P Size and predictive factors of microscopic tumor extension in locally advanced non-small cell lung cancer. Pract Radiat Oncol. 2021; 10.1016/j.prro.2021.05.006. 10.1016/j.prro.2021.05.006 34126295

[20] Whitfield GA, Kennedy SR, Djoukhadar IK, Jackson A. Imaging and target volume delineation in glioma. Clin Oncol (R Coll Radiol). 2014; 26(7):364-376. 10.1016/j.clon.2014.04.026. 10.1016/j.clon.2014.04.026 24824451

[21] Castellano A, Bailo M, Cicone F, Carideo L, Quartuccio N, Mortini P, et al. Advanced imaging techniques for radiotherapy planning of gliomas. Cancers (Basel). 2021; 13(5). 10.3390/cancers13051063. 10.3390/cancers13051063 PMC7959155 33802292

[22] Mair DB, Ames HM, Li R. Mechanisms of invasion and motility of high-grade gliomas in the brain. Mol Biol Cell. 2018; 29(21):2509-2515. 10.1091/mbc.E18-02-0123. 10.1091/mbc.E18-02-0123 PMC6254577 30325290

[23] Wank M, Schilling D, Schmid TE, Meyer B, Gempt J, Barz M, et al. Human glioma migration and infiltration properties as a target for personalized radiation medicine. Cancers (Basel). 2018; 10(11). 10.3390/cancers10110456. 10.3390/cancers10110456 PMC6266328 30463322

[24] Brandes AA, Tosoni A, Franceschi E, Sotti G, Frezza G, Amistà P, Morandi L, Spagnolli F, Ermani M Recurrence pattern after temozolomide concomitant with and adjuvant to radiotherapy in newly diagnosed patients with glioblastoma: correlation With MGMT promoter methylation status. J Clin Oncol. 2009; 27(8):1275-1279. 10.1200/jco.2008.19.4969. 10.1200/JCO.2008.19.4969 19188675

[25] Minniti G, Amelio D, Amichetti M, Salvati M, Muni R, Bozzao A, Lanzetta G, Scarpino S, Arcella A, Enrici RM. Patterns of failure and comparison of different target volume delineations in patients with glioblastoma treated with conformal radiotherapy plus concomitant and adjuvant temozolomide. Radiother Oncol. 2010; 97(3):377-381. /10.1016/j.radonc.2010.08.020. 10.1016/j.radonc.2010.08.020 20855119

[26] Choi SH, Kim JW, Chang JS, Cho JH, Kim SH, Chang JH, Suh CO Impact of including peritumoral edema in radiotherapy target volume on patterns of failure in glioblastoma following temozolomide-based chemoradiotherapy. Sci Rep. 2017; 7:42148. 10.1038/srep42148. 10.1038/srep42148 PMC5296913 28176884

[27] Liang HT, Chen WY, Lai SF, Su MY, You SL, Chen LH, et al. The extent of edema and tumor synchronous invasion into the subventricular zone and corpus callosum classify outcomes and radiotherapy strategies of glioblastomas. Radiother Oncol. 2017; 125(2):248-257. 10.1016/j.radonc.2017.09.024. 10.1016/j.radonc.2017.09.024 29056290

[28] Cuddapah VA, Robel S, Watkins S, Sontheimer H. A neurocentric perspective on glioma invasion. Nat Rev Neurosci. 2014; 15(7):455-465. 10.1038/nrn3765. 10.1038/nrn3765 PMC5304245 24946761

[29] Sharifi G, Pajavand AM, Nateghinia S, Meybodi TE, Hasooni H. Glioma migration through the corpus callosum and the brainstem detected by diffusion and magnetic resonance imaging: initial findings. Front Hum Neurosci. 2019; 13:472. 10.3389/fnhum.2019.00472. 10.3389/fnhum.2019.00472 PMC7052521 32161524

[30] D'Souza S, Ormond DR, Costabile J, Thompson JA. Fiber-tract localized diffusion coefficients highlight patterns of white matter disruption induced by proximity to glioma. PLoS One. 2019; 14(11):e0225323. 10.1371/journal.pone.0225323. 10.1371/journal.pone.0225323 PMC6874090 31751402

[31] Wang X, Zhou C, Wang L, Wang Y, Jiang T. Motor cortex gliomas induces microstructural changes of large fiber tracts revealed by TBSS. Sci Rep. 2020; 10(1):16900. 10.1038/s41598-020-73746-1. 10.1038/s41598-020-73746-1 PMC7547011 33037275

[32] Unkelbach J, Menze BH, Konukoglu E, Dittmann F, Le M, Ayache N, et al. Radiotherapy planning for glioblastoma based on a tumor growth model: improving target volume delineation. Phys Med Biol. 2014; 59(3):747-770. 10.1088/0031-9155/59/3/747. 10.1088/0031-9155/59/3/747 PMC12895353 24440875

