# Supplementary material for: Clinicopathologic analysis of microscopic tumor extension in glioma for external beam radiotherapy planning
Source: BMC Med. 2021 Nov 17;19:269. doi: 10.1186/s12916-021-02143-w (PMC8597244; doi:10.1186/s12916-021-02143-w)
Supplement: Supplementary file 1 — Additional file 1: Table S1. Macroscopic tumor size before and after fixation, corresponding area retraction ratio for each case. [file 12916_2021_2143_MOESM1_ESM.docx]

**Additional file 1**

**Table S1.** Macroscopic tumor size before and after fixation, corresponding area retraction ratio for each case

| Case no. | Tumor grade | D_long_ | D_short_ | d_long_ | d_short_ | Area retraction |
| --- | --- | --- | --- | --- | --- | --- |
| 1 | II | 3.42 | 2.70 | 3.08 | 2.63 | 0.88 |
| 2 | II | 2.29 | 1.45 | 2.23 | 1.38 | 0.93 |
| 3 | II | 2.39 | 1.70 | 2.39 | 1.70 | 1.00 |
| 4 | II | 5.01 | 4.12 | 4.74 | 3.78 | 0.87 |
| 5 | II | 4.08 | 1.98 | 3.72 | 1.66 | 0.76 |
| 6 | II | 2.69 | 1.77 | 2.44 | 1.52 | 0.78 |
| 7 | II | 3.44 | 2.59 | 3.44 | 2.59 | 1.00 |
| 8 | II | 1.83 | 1.59 | 1.45 | 1.37 | 0.68 |
| 9 | III | 5.63 | 4.92 | 5.09 | 4.25 | 0.78 |
| 10 | III | 1.80 | 1.52 | 1.56 | 1.29 | 0.74 |
| 11 | III | 2.49 | 2.15 | 2.06 | 1.83 | 0.70 |
| 12 | III | 3.89 | 2.99 | 3.89 | 2.99 | 1.00 |
| 13 | III | 4.14 | 3.21 | 4.14 | 3.21 | 1.00 |
| 14 | III | 4.42 | 4.19 | 3.98 | 3.63 | 0.78 |
| 15 | III | 4.08 | 2.73 | 3.94 | 2.67 | 0.94 |
| 16 | III | 4.81 | 3.90 | 4.67 | 3.58 | 0.89 |
| 17 | III | 4.88 | 4.02 | 4.88 | 4.02 | 1.00 |
| 18 | III | 4.97 | 4.82 | 4.68 | 4.51 | 0.88 |
| 19 | III | 2.90 | 1.61 | 2.77 | 1.43 | 0.85 |
| 20 | III | 6.58 | 6.14 | 5.83 | 5.46 | 0.79 |
| 21 | III | 4.33 | 3.03 | 4.33 | 3.03 | 1.00 |
| 22 | III | 1.80 | 1.67 | 1.80 | 1.67 | 1.00 |
| 23 | III | 4.41 | 4.30 | 4.09 | 3.86 | 0.83 |
| 24 | III | 6.74 | 4.35 | 6.28 | 4.01 | 0.86 |
| 25 | III | 3.14 | 2.83 | 3.14 | 2.83 | 1.00 |
| 26 | IV | 1.73 | 1.51 | 1.68 | 1.38 | 0.89 |
| 27 | IV | 2.15 | 1.78 | 1.85 | 1.34 | 0.65 |
| 28 | IV | 3.69 | 2.92 | 3.02 | 2.49 | 0.70 |
| 29 | IV | 2.33 | 1.80 | 2.24 | 1.68 | 0.90 |
| 30 | IV | 4.94 | 4.18 | 4.94 | 4.18 | 1.00 |
| 31 | IV | 5.69 | 3.59 | 5.55 | 3.47 | 0.94 |
| 32 | IV | 3.14 | 2.75 | 2.44 | 2.16 | 0.61 |
| 33 | IV | 4.19 | 3.14 | 3.57 | 2.78 | 0.75 |
| 34 | IV | 2.77 | 2.48 | 2.77 | 2.48 | 1.00 |
| 35 | IV | 4.60 | 4.51 | 4.13 | 4.03 | 0.80 |
| 36 | IV | 4.83 | 3.45 | 4.83 | 3.45 | 1.00 |
| 37 | IV | 4.67 | 3.15 | 4.67 | 3.15 | 1.00 |
| 38 | IV | 3.27 | 3.06 | 2.88 | 2.47 | 0.71 |

***Abbreviations*:** D_long_ = long diameter on transverse plane before formalin fixation; D_short_ = short diameter on transverse plane before formalin fixation; d_long_ = long diameter on transverse plane after formalin fixation; d_short_ = short diameter on transverse plane after formalin fixation; Area retraction = tissue transverse plane reduction after formalin fixation, which was calculated according to the formula (d_long_ × d_short_) / (D_long_ × D_short_)
