# Supplementary material for: Clinicopathologic analysis of microscopic tumor extension in glioma for external beam radiotherapy planning
Source: BMC Med. 2021 Nov 17;19:269. doi: 10.1186/s12916-021-02143-w (PMC8597244; doi:10.1186/s12916-021-02143-w)
Supplement: Supplementary file 2 — Additional file 2:. Molecular testing methods. (a) MGMT promoter methylation. (b) IDH1 mutations. (c) Detection of 1p/19q co-deletion. [file 12916_2021_2143_MOESM2_ESM.docx]

**Additional file 2**

**Molecular testing methods**

**(a) MGMT promoter methylation**

The methylation status of the MGMT promoter was assessed using DNA pyrosequencing. Bisulfite modification of the DNA was performed using the EpiTect Kit (Qiagen). Two primers were used to amplify the MGMT promoter region forward: 5′-GTTTYGGATATGTTGGGATA-3′ and 5′-biotin-ACCCAAACACTCACCAAATC-3′. The PCR analysis was performed in duplicate in 40 μL reaction volume, containing 0.5 μL of 10 μM each primer, 4 μL 10 × buffer, 3.2 μL of 2.5 μM dNTPs, 2.5 U hotstart Taq (Takara, Madison, WI) and 2 μL of 10 μM bisulfite-treated DNA. The PCR conditions were: 95℃ for 3 minutes; 40 cycles of 95℃ for 15 seconds, 52℃ for 30 seconds, 72℃ for 30 seconds; and 72℃ for 5 minutes (ABI PCR system 9700). DNA was purified from the total PCR products using QIAamp DNA Mini Kit (Qiagen) and subjected to pyrosequencing (PyroMark Q96 ID System [Qiagen]) using the primer 5′- GGATATGTTGGGATAGT-3′ according to the manufacturer’s instructions. The methylation values obtained were averaged across the seven CpG loci tested within the MGMT promoter. The samples were considered MGMT methylated with an average methylation of > 10%.

**(b) IDH1 mutations**

IDH1 mutations were identified using DNA pyrosequencing. A QIAamp DNA Mini Kit (Qiagen) was used to isolate genomic DNA from frozen tumor tissue samples. The genomic region spanning the wild-type R132 of IDH1 was analyzed by amplifying a 75-base pair (bp) fragment with the following primers: 5′-GCTTGTGAGTGGATGGGTAAAAC-3′ and 5′-biotin-TTGCCAACATGACTTACTTGATC-3′. Duplicate PCR analyses were performed in 40 μL reaction tubes containing 1 μL each of 10 μM forward and reverse primers, 4 μL of 10 × buffer, 3.2 μL of 2.5 mM dNTPs, 2.5 U HotStar Taq (Takara), and 2 μ L of 10 μM DNA. The PCR conditions were as follows: 95°C for 3 minutes, 50 cycles at 95°C for 15 seconds, 56°C for 20 seconds, 72°C for 30 seconds, and then 72°C for 5 minutes (ABI PCR System 9700; Applied Biosystems). Single-stranded DNA was purified from the PCR products and pyrosequenced with a PyroMark Q96 ID System (Qiagen) using a 5′-TGGATGGGTAAAACCT-3′ primer and an EpiTect Bisulfite Kit (Qiagen).

**(c) Detection of 1p/19q co-deletion**

Deletion of chromosomal arms 1p and 19q was tested by use of fluorescence in-situ hybridisation (FISH). For 1p, probes to 1p36 (D1S32) and centromere 1 (pUC1.77) were used. For the assessment of 19q, probes were used directed to 19p (equivalent amounts of BAC 959O6, 957I1, and 153P24) and 19q (BAC 426G3). A Zeiss Axioplan microscope (Zeiss, Jena, Germany) equipped with single-pass, dual-pass, and triple-pass filters (4,6-diamidino-2-phenylindole [DAPI]; fluorescin isothiocyanate [FITC]; and tetramethylrho-damine isothiocyanate [TRITC]) was used to count the number of FISH signals for each locus-specific FISH probe. Sixty nonoverlapping nuclei were enumerated per hybridization. Ratios were calculated for 1p versus centromere 1 or 19q versus 19p by dividing the number of signals of the marker by the number of signals of the reference; a ratio of less than 0.80 was considered as allelic loss. If a borderline ratio was obtained (0.75 to 0.90), spots in 200 nuclei were counted.
