# Supplementary material for: Clinicopathologic analysis of microscopic tumor extension in glioma for external beam radiotherapy planning
Source: BMC Med. 2021 Nov 17;19:269. doi: 10.1186/s12916-021-02143-w (PMC8597244; doi:10.1186/s12916-021-02143-w)

**Additional file 3**

**Fig. S1**

(a) Correlation between radiologic tumor size measured on MR imaging and macroscopic tumor size on enhancing gliomas

**
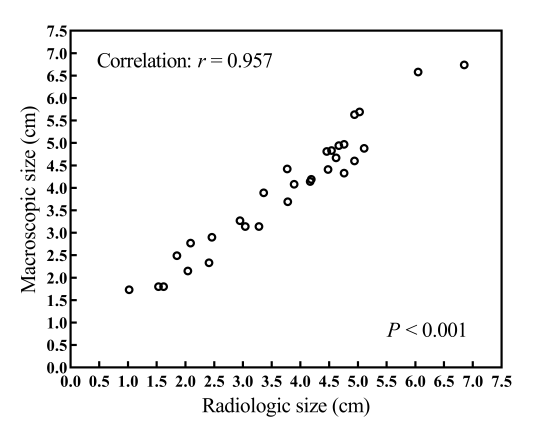
**

(b) Correlation between radiologic tumor size measured on MR imaging and histologic tumor size on enhancing gliomas


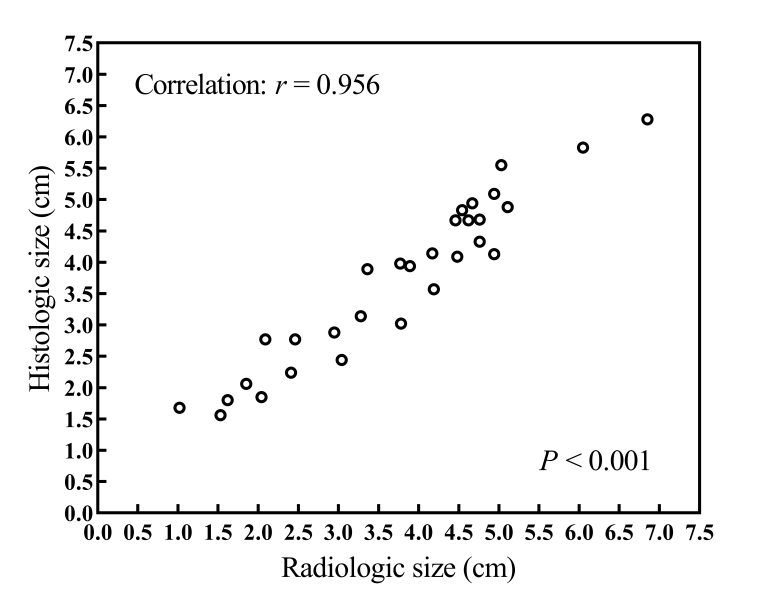


(c) Correlation between radiologic tumor size measured on MR imaging and macroscopic tumor size on non-enhancing gliomas


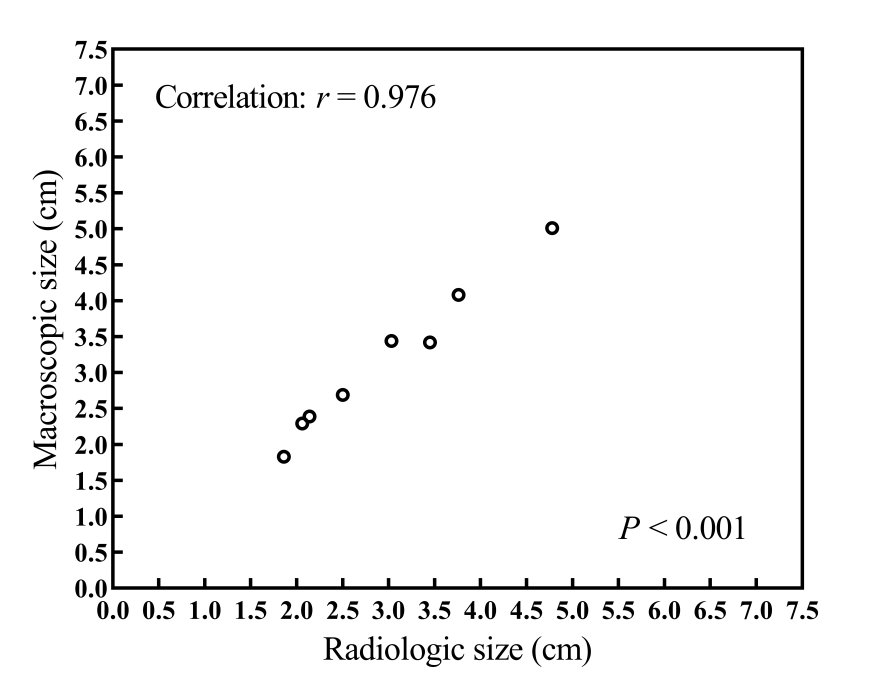


(d) Correlation between radiologic tumor size measured on MR imaging and histologic tumor size on non-enhancing gliomas


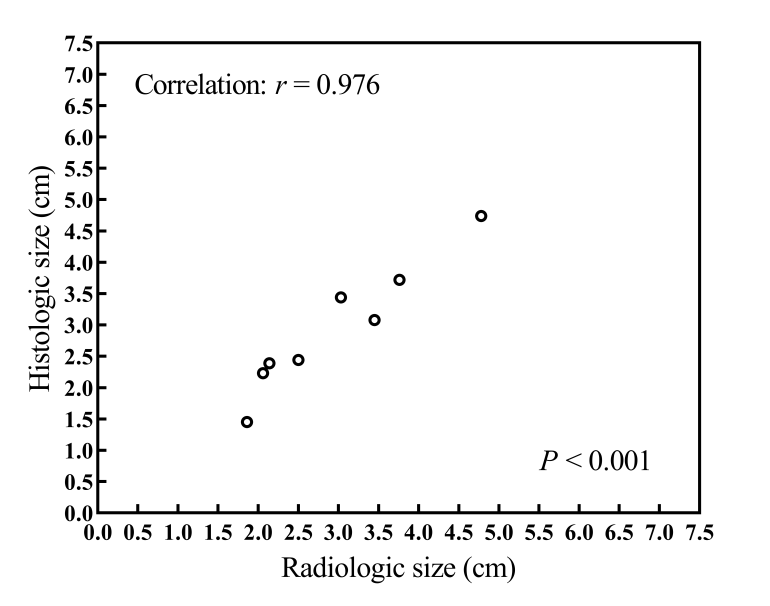

Supplement: Supplementary file 3 — Additional file 3: Fig. S1. (a) Correlation between radiologic tumor size measured on MR imaging and macroscopic tumor size on enhancing gliomas. (b) Correlation between radiologic tumor size measured on MR imaging and histologic tumor size on enhancing gliomas. (c) Correlation between radiologic tumor size measured on MR imaging and macroscopic tumor size on non-enhancing gliomas. (d) Correlation between radiologic tumor size measured on MR imaging and histologic tumor size on non-enhancing gliomas. [file 12916_2021_2143_MOESM3_ESM.docx]
