# Supplementary material for: Clinicopathologic analysis of microscopic tumor extension in glioma for external beam radiotherapy planning
Source: BMC Med. 2021 Nov 17;19:269. doi: 10.1186/s12916-021-02143-w (PMC8597244; doi:10.1186/s12916-021-02143-w)
Supplement: Supplementary file 4 — Additional file 4: Fig. S2. Histogram analysis showing the microscopic extension in different subgroups. (a) Tumor grade. (b) MGMT promoter methylation status. (c) IDH mutation status. (d) 1p/19q co-deletion status. [file 12916_2021_2143_MOESM4_ESM.docx]

**Additional file 4**

**Fig. S2**

Histogram analysis showing the microscopic extension in different subgroups. **(a)** Tumor grade. **(b)** MGMT promoter methylation status. **(c)** IDH mutation status. **(d)** 1p/19q co-deletion status.

**
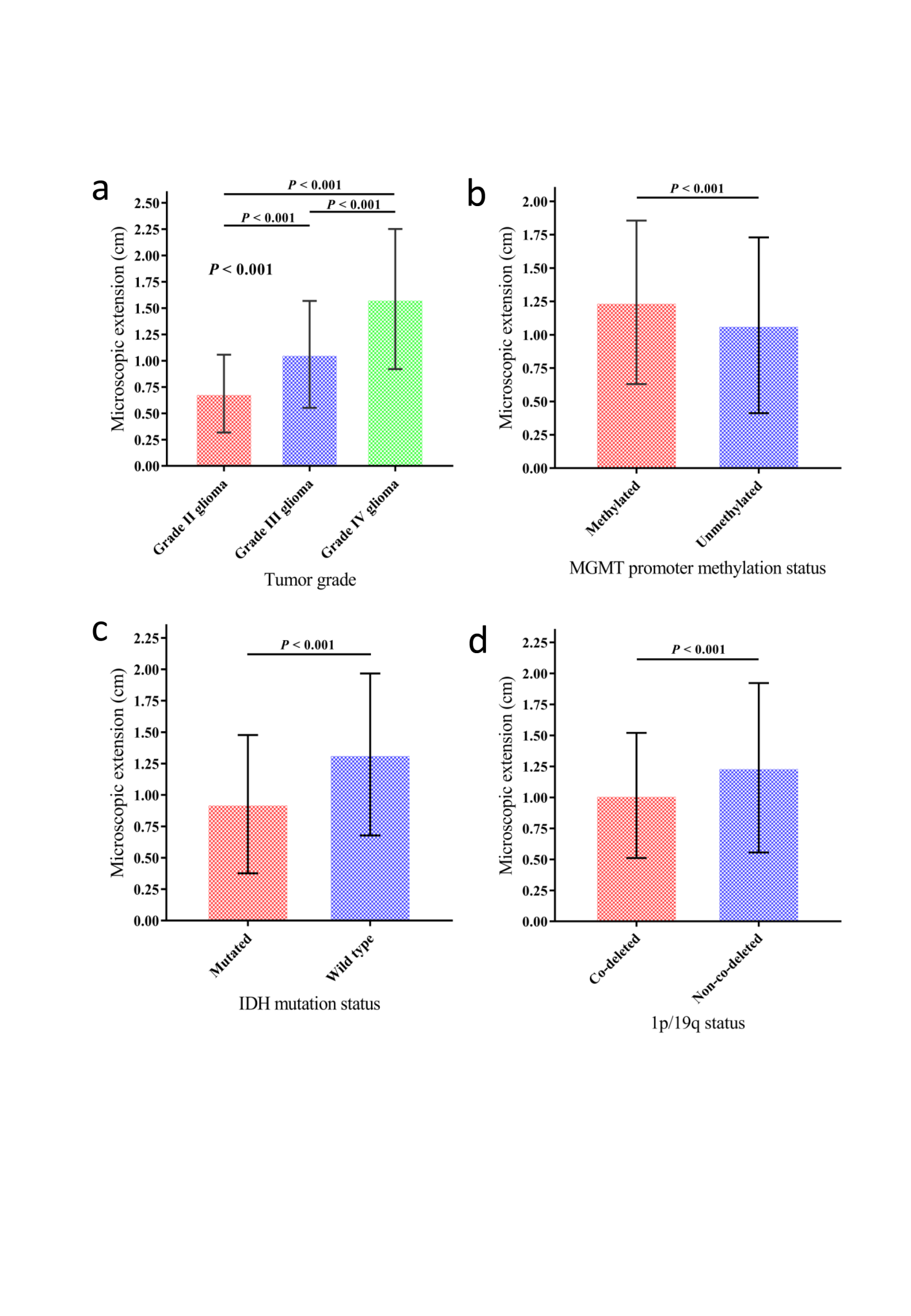
**
