# Supplementary material for: Clinicopathologic analysis of microscopic tumor extension in glioma for external beam radiotherapy planning
Source: BMC Med. 2021 Nov 17;19:269. doi: 10.1186/s12916-021-02143-w (PMC8597244; doi:10.1186/s12916-021-02143-w)
Supplement: Supplementary file 5 — Additional file 5: Fig. S3. Histogram analysis showing the microscopic extension in different molecular groups. [file 12916_2021_2143_MOESM5_ESM.docx]

**Additional file 5**

**Fig. S3**

Histogram analysis showing the microscopic extension in different molecular groups


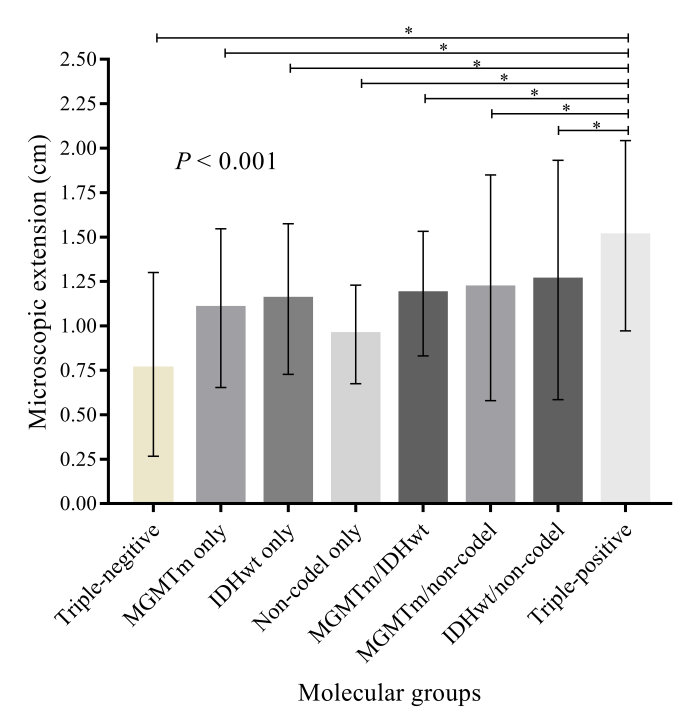


^*^ *P* < 0.05.

***Abbreviation:*** Triple-negitive = tumor which had both MGMT unmethylated and IDH mutated plus 1p/19q co-deleted status; MGMT_m_ only = tumor which had only MGMT methylated status; IDH_wt_ only = tumor which had only IDH wild-type status; Non-codel only = tumor which had only 1p/19q non-co-deleted status; MGMT_m_/IDH_wt_ = tumor which had both MGMT methylated and IDH wild-type status; MGMT_m_/non-codel = tumor which had both MGMT methylated and 1p/19q non-co-deleted status; IDH_wt_/non-codel = tumor which had both IDH wild-type and 1p/19q non-co-deleted status; Triple-positive = tumor which had both MGMT methylated and IDH wild-type plus 1p/19q non-co-deleted status
