# Supplementary material for: Clinicopathologic analysis of microscopic tumor extension in glioma for external beam radiotherapy planning
Source: BMC Med. 2021 Nov 17;19:269. doi: 10.1186/s12916-021-02143-w (PMC8597244; doi:10.1186/s12916-021-02143-w)
Supplement: Supplementary file 6 — Additional file 6: Table S2. The invasion mode between different grades of gliomas. [file 12916_2021_2143_MOESM6_ESM.docx]

**Additional file 6**

**Table S2**

The invasion mode between different grades of gliomas

| Invasion mode | LGG | | HGG | | Total | | *P*^**^ |
| --- | --- | --- | --- | --- | --- | --- | --- |
|  | *n*^*^ | (%) | *n*^*^ | (%) | *n*^*^ | (%) |  |
| Direct extension | 133 | (82) | 417 | (64) | 550 | (68) | < 0.001 |
| Perineural spread | 7 | (4) | 88 | (13) | 95 | (12) | 0.001 |
| Subpial spread | 8 | (5) | 82 | (13) | 90 | (11) | 0.006 |
| Perivascular extension | 14 | (9) | 65 | (10) | 79 | (10) | 0.610 |

^*^ Number of slides presenting this criterion.

^**^*P* value according to the *Chi-squared* test*.*

***Abbreviation:*** LGG = low-grade glioma; HGG = high-grade glioma
