# Supplementary material for: Clinicopathologic analysis of microscopic tumor extension in glioma for external beam radiotherapy planning
Source: BMC Med. 2021 Nov 17;19:269. doi: 10.1186/s12916-021-02143-w (PMC8597244; doi:10.1186/s12916-021-02143-w)
Supplement: Supplementary file 7 — Additional file 7: Table S3. (a) ME distribution for grade II glioma in triple-positive molecular group. (b) ME distribution for grade III glioma in triple-positive molecular group. (c) ME distribution for grade IV glioma in triple-positive molecular group. Fig. S4. Cumulative distribution of ME for different grades gliomas in triple-positive molecular group. [file 12916_2021_2143_MOESM7_ESM.docx]

**Additional file 7**

**Table S3**

(a) ME distribution for grade II glioma in triple-positive molecular group

| ME (cm) | No. | Cumulative | No. % | Cumulative % |
| --- | --- | --- | --- | --- |
| 0.00 | 5 | 5 | 6.85 | 6.85 |
| 0.20 | 2 | 7 | 2.74 | 9.59 |
| 0.40 | 3 | 10 | 4.11 | 13.70 |
| 0.50 | 3 | 13 | 4.11 | 17.81 |
| 0.60 | 4 | 17 | 5.48 | 23.29 |
| 0.70 | 3 | 20 | 4.11 | 27.40 |
| 0.80 | 10 | 30 | 13.70 | 41.10 |
| 0.90 | 10 | 40 | 13.70 | 54.80 |
| 1.00 | 25 | 65 | 34.25 | 89.04 |
| 1.30 | 1 | 66 | 1.37 | 90.41 |
| 1.50 | 4 | 70 | 5.48 | 95.89 |
| 1.70 | 2 | 72 | 2.74 | 98.63 |
| 1.80 | 1 | 73 | 1.37 | 100 |

(b) ME distribution for grade III glioma in triple-positive molecular group

| ME (cm) | No. | Cumulative | No. % | Cumulative % |
| --- | --- | --- | --- | --- |
| 0.00 | 7 | 7 | 10.14 | 10.14 |
| 0.20 | 2 | 9 | 2.90 | 13.04 |
| 0.30 | 1 | 10 | 1.45 | 14.49 |
| 0.50 | 2 | 12 | 2.90 | 17.39 |
| 0.70 | 1 | 13 | 1.45 | 18.84 |
| 0.80 | 3 | 16 | 4.35 | 23.19 |
| 0.90 | 8 | 24 | 11.59 | 34.89 |
| 1.00 | 5 | 29 | 7.25 | 42.03 |
| 1.20 | 10 | 39 | 14.49 | 56.52 |
| 1.30 | 2 | 41 | 2.90 | 59.42 |
| 1.40 | 9 | 50 | 13.04 | 72.46 |
| 1.50 | 11 | 61 | 15.94 | 88.41 |
| 1.90 | 4 | 65 | 5.80 | 94.20 |
| 2.00 | 1 | 66 | 1.45 | 95.65 |
| 2.10 | 1 | 67 | 1.45 | 97.10 |
| 2.30 | 2 | 69 | 2.90 | 100 |

(c) ME distribution for grade IV glioma in triple-positive molecular group

| ME (cm) | No. | Cumulative | No. % | Cumulative % |
| --- | --- | --- | --- | --- |
| 0.00 | 11 | 11 | 8.73 | 8.73 |
| 1.40 | 1 | 12 | 0.79 | 9.52 |
| 1.50 | 6 | 18 | 4.76 | 14.29 |
| 1.60 | 9 | 27 | 7.14 | 21.43 |
| 1.70 | 7 | 34 | 5.56 | 26.98 |
| 1.80 | 33 | 67 | 26.19 | 53.17 |
| 1.90 | 46 | 113 | 36.51 | 89.68 |
| 2.00 | 6 | 119 | 4.76 | 94.44 |
| 2.50 | 1 | 120 | 0.79 | 95.24 |
| 2.60 | 4 | 124 | 3.17 | 98.41 |
| 2.70 | 1 | 125 | 0.79 | 99.21 |
| 2.80 | 1 | 126 | 0.79 | 100 |

***Abbreviation:*** ME = Microscopic extension; Triple-positive = tumor which had both MGMT methylated and IDH wild-type plus 1p/19q non-co-deleted status; No = Number of slides

**Fig. S4**

Cumulative distribution of ME for different grades gliomas in triple-positive molecular group


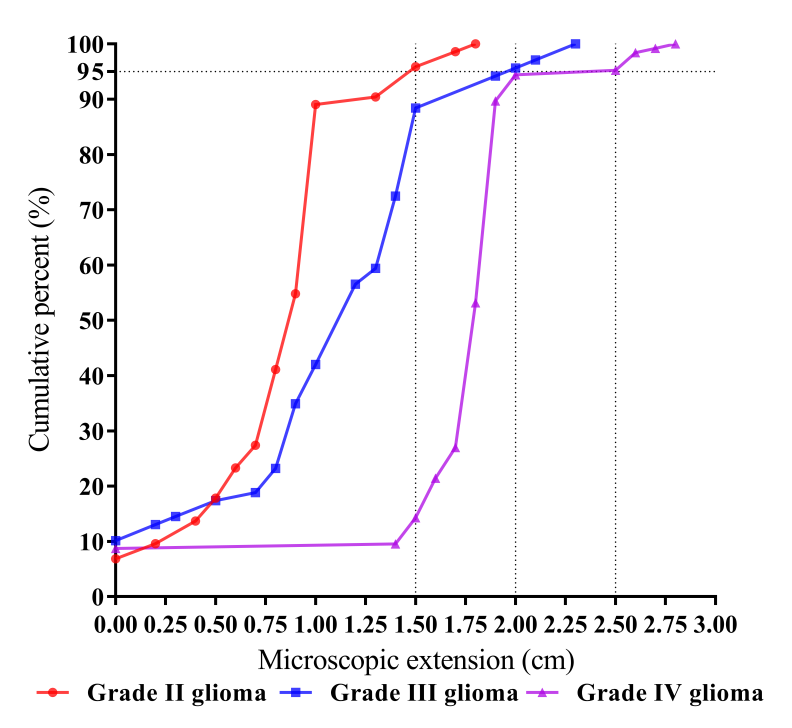


***Abbreviation:*** ME = Microscopic extension; Triple-positive = tumor which had both MGMT methylated and IDH wild-type plus 1p/19q non-co-deleted status.
