# Supplementary material for: Clinicopathologic analysis of microscopic tumor extension in glioma for external beam radiotherapy planning
Source: BMC Med. 2021 Nov 17;19:269. doi: 10.1186/s12916-021-02143-w (PMC8597244; doi:10.1186/s12916-021-02143-w)

**Additional file 8**

**CTV performance validation**

**Methods**

To validate the efficacy and safety of the CTV margin determined based on ME (CTV_ME_), we retrospectively collected 139 patients with glioma who underwent radiotherapy at Shandong Cancer Hospital. The details of the patient enrollment, radiotherapy planning and follow-up are displayed as following. Patients were divided into CTV_ME_ radiotherapy group and non-CTV_ME_ radiotherapy group according to their CTV margin size. The primary endpoint was overall survival (OS) (the date from surgery to the date of death or the last follow-up), and secondary endpoints included both progression-free survival (PFS) (the date from surgery to either first documented progression or death) and adverse events, which were assessed according to Common Toxicity Criteria Adverse Events version 5.0.

**Patient enrollment.** This retrospective study was reviewed and approved by Shandong Cancer Hospital and Institute. Written informed consent was obtained from patients prior to the commencement of the study. The relevant demographic and clinical data on patients were obtained and identified from the institutional electronic medical record system. All patients met the following eligibility criteria: (a) age ≥ 18 years and less than 65 years; (b) pathologically confirmed glioma; and (c) Karnofsky performance status ≥ 80. The exclusion criteria included: (a) a medical history of brain chemoradiotherapy; and (b) tumor located in the brainstem. All patients were treated with radiotherapy plus concomitant daily TMZ (75mg/m^2^/d), followed by 6 cycles of adjuvant TMZ (150–200 mg/m^2^ for 5/28 days).

**Radiotherapy planning.** Radiotherapy started within 4-6 weeks of surgery and consisted of fractionated focal irradiation, at the dose of 54 Gy for LGG and 60 Gy for HGG delivered in 1.8-2.0 Gy per fraction for a total of 30 fractions. Radiation treatment planning was performed with the Varian Eclipse Treatment Planning System. Target volumes were delineated according to co-registration of postoperative CT and MR imaging obtained with patients in the treatment position. GTV included the surgical cavity plus any residual enhancing tumor on T1-weighted MR imaging (in non-enhancing gliomas, consideration may be given to include FLAIR abnormality in the GTV). How far to extend the CTV beyond the GTV is mainly left to the discretion of the physician. In general, the CTV was the GTV with an extended edge of 0.5-2 cm for LGG and 1-3 cm for HGG, adjusted to anatomical borders. The CTV was expanded by 3-5 mm to create the respective PTV. Organs at risk, such as the brain stem, lens, optic chiasm, optic nerve and pituitary was ≤54 Gy, ≤7 Gy, ≤56 Gy, ≤55 Gy and ≤50 Gy, respectively.

**Follow-up.** MR imaging was repeated before radiotherapy, before the first cycle of adjuvant TMZ, and thereafter every 3-6 months. Tumor progression was identified by both the physician and the radiologist. Response criteria as defined by Macdonald et al. were used. Tumor progression was defined by an increase in tumor size more than 25% or by the presence of a new lesion on imaging. Radiological progression had to be confirmed at two different MR imaging evaluations (at least 2 months apart). Adverse events were recorded and graded according to the Common Terminology Criteria for Adverse Events version 5.0.

**Statistical analysis**

The patient survival rates were determined by Kaplan-Meier curves and survival curves were analyzed by the *log-rank* test. Statistical analyses were performed using SPSS Statistical software version 22.0 (IBM Armonk, New York, USA) and R version 3.4.0 (http://www. R-project.org/), and statistical significance was set at *P* < 0.05.

**Results**

**Validation cohort**

Between May 2017 and March 2021, 139 patients with glioma (mean age: 45.87 years, range: 18-64 years; 53% male) were enrolled. The patients included 51 grade III gliomas and 88 grade IV gliomas. Grade II gliomas were excluded because none of the patients were eligible for CTV_ME_ radiotherapy. Characteristics of patients are listed in **Additional file 8: Table S4**. At a median follow-up of 18.4 months, 39 patients (39/139, 28%) had progression or death. CTV_ME_ radiotherapy group had a slightly higher median OS (18.9 months vs. 18.1 months, *P* = 0.944, hazard ratio [HR] = 0.966, 95% confidence interval [CI], 0.370 to 2.521) and median PFS (18.4 months vs. 15.7 months, *P* = 0.168, HR = 0.637, 95% CI, 0.333 to 1.217) than non-CTV_ME_ radiotherapy group, but the difference was not statistically significant (**Additional file 8: Fig. S5a,b**). These were also found for different grades of gliomas (**Additional file 8: Fig. S5c-f**). Non-CTV_ME_ radiotherapy group had more acute toxicities; the most frequent and serious toxicities were cognitive change and peripheral neuropathy. Serious immediate and late toxicities related to radiotherapy were uncommon.

**Table S4**

Baseline characteristics of validation cohort

| Characteristic | CTV_ME_ group | | non-CTV_ME_ group | | Total | | *P*^*^ |
| --- | --- | --- | --- | --- | --- | --- | --- |
|  | *n* | (%) | *n* | (%) | *n* | (%) |  |
| Patients | 67 | (48) | 72 | (52) | 139 | (100) |  |
| Age (years) |  | | | | | | 0.929 |
| Mean (SD) | 45.78 | (11.91) | 45.96 | (12.03) | 45.87 | (11.93) |  |
| Median (IQR) | 49 | (35-55) | 49.5 | (36-55.75) | 49 | (36-55) |  |
| Gender |  | | | | | | 0.949 |
| Male | 35 | (52) | 38 | (53) | 73 | (53) |  |
| Female | 32 | (48) | 34 | (47) | 66 | (47) |  |
| KPS |  | | | | | | 0.204 |
| 80-90 | 37 | (55) | 32 | (44) | 69 | (50) |  |
| ≥ 90 | 30 | (45) | 40 | (56) | 70 | (50) |  |
| Tumor grade |  | | | | | | 0.395 |
| Grade III | 27 | (40) | 24 | (33) | 51 | (37) |  |
| Grade IV | 40 | (60) | 48 | (67) | 88 | (63) |  |
| Lesion site |  | | | | | | 0.916 |
| Frontal lobe | 26 | (39) | 28 | (39) | 54 | (39) |  |
| Temporal lobe | 11 | (16) | 10 | (14) | 21 | (15) |  |
| Occipital lobe | 18 | (27) | 18 | (25) | 36 | (26) |  |
| Others | 12 | (18) | 16 | (22) | 28 | (20) |  |
| Extent of resection |  | | | | | | 0.805 |
| Subtotal resection | 37 | (55) | 43 | (60) | 80 | (58) |  |
| Near-total resection | 24 | (36) | 22 | (31) | 46 | (33) |  |
| Gross total resection | 6 | (9) | 7 | (10) | 13 | (9) |  |
| MGMT methylation status |  | | | | | | 0.499 |
| Unmethylated | 25 | (37) | 23 | (32) | 48 | (35) |  |
| Methylated | 29 | (43) | 29 | (40) | 58 | (42) |  |
| Losing | 13 | (19) | 20 | (28) | 33 | (24) |  |
| IDH mutation |  | | | | | | 0.376 |
| Mutated | 12 | (18) | 15 | (21) | 27 | (19) |  |
| Wild type | 42 | (63) | 37 | (51) | 79 | (57) |  |
| Losing | 13 | (19) | 20 | (28) | 33 | (24) |  |
| 1p/19q co-deletion |  | | | | | | 0.439 |
| Co-deleted | 15 | (22) | 17 | (24) | 32 | (23) |  |
| Non-co-deleted | 39 | (58) | 35 | (49) | 74 | (53) |  |
| Losing | 13 | (19) | 20 | (28) | 33 | (24) |  |

^*^*P* value according to the *Student’s t-*test or *Chi-squared* test*.*

***Abbreviation:*** CTV = Clinical target volume; ME = Microscopic extension; CTV_ME_ = CTV margin which is determined based on ME; non-CTV_ME_ = CTV margin which is not determined based on ME; SD = Standard Deviation; IQR = Interquartile range; KPS = Karnofsky performance status; MGMT = O^6^-methylguanine-DNA-methyltransferase; IDH = isocitrate dehydrogenase; 1p/19q co-deletion = the co-deletion of chromosome arms 1p and 19q

**Fig. S5**

Comparison of overall survival and progression-free survival in all patients and different grades of gliomas. (a, b) Overall; (c, d) Grade III gliomas; (e, f) Grade IV gliomas. CTV_ME_ = CTV margin which is determined based on ME; non-CTV_ME_ = CTV margin which is not determined based on ME.


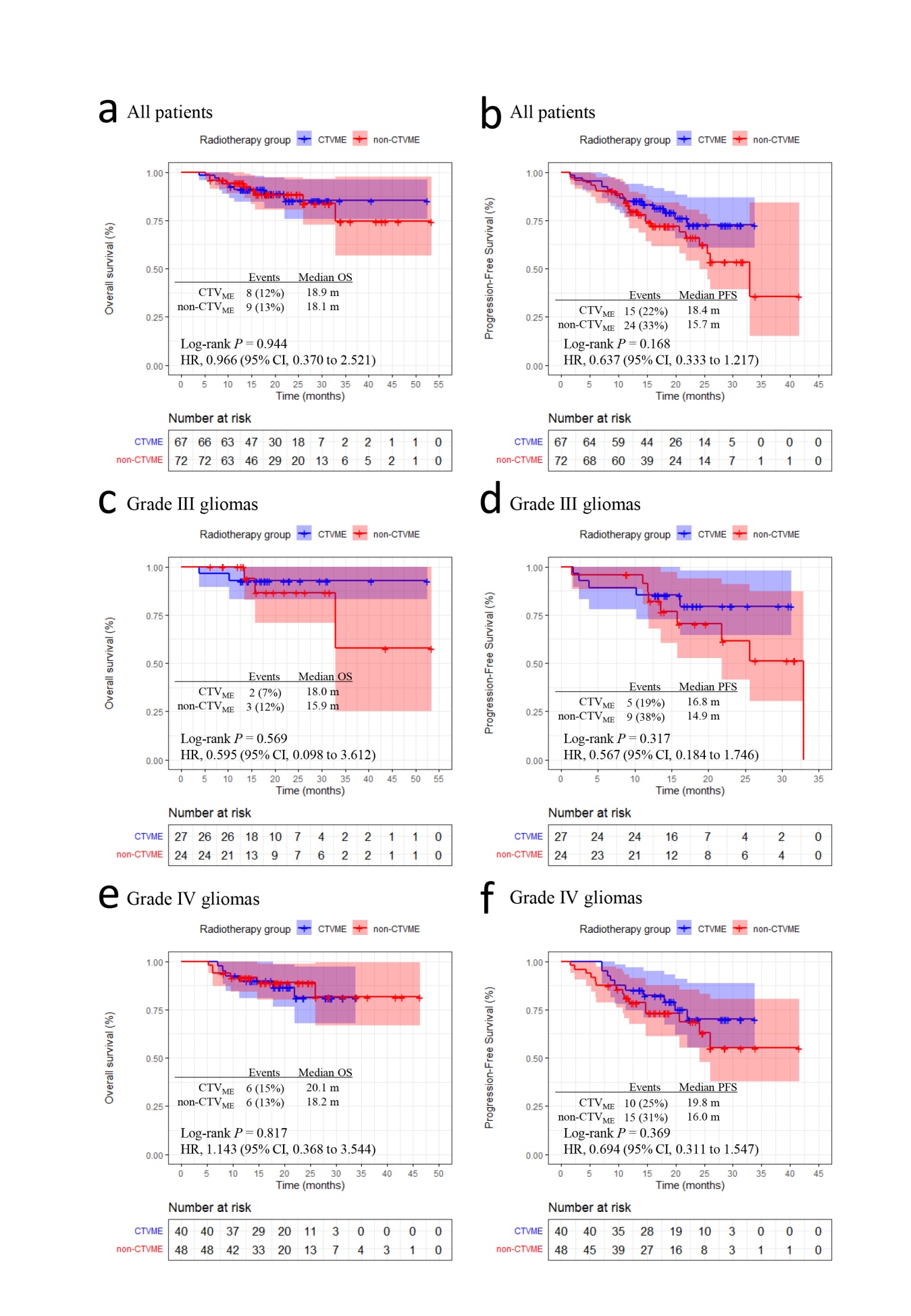

Supplement: Supplementary file 8 — Additional file 8: CTV performance validation. Table S4. Baseline characteristics of validation cohort. Fig. S5. Comparison of overall survival and progression-free survival in all patients and different grades of gliomas. [file 12916_2021_2143_MOESM8_ESM.docx]
